# First-principles investigation of aspirin, paracetamol, and ibuprofen adsorption on triquinoxalinylene and benzoquinone-based covalent organic framework

**DOI:** 10.1038/s41598-025-25834-3

**Published:** 2026-04-10

**Authors:** Sami Bawazeer

**Affiliations:** Department of Pharmaceutical Sciences, Faculty of Pharmacy, Umm Al-Qura University, Makkah, Saudi Arabia

**Keywords:** TQBQ-COF, Drug adsorption, First-principles, Removal, Chemistry, Materials science

## Abstract

Pharmaceutical contaminants such as aspirin (ASP), paracetamol (PAR), and ibuprofen (IBU) present increasing risks to environmental and human health, necessitating efficient detection and removal strategies. In this study, we performed a comprehensive first-principles investigation of their adsorption on a triquinoxalinylene and benzoquinone-based covalent organic framework (TQBQ-COF). The geometries were optimized and the adsorption complexes were characterized using density functional theory. The results reveal that all three drug molecules are effectively accommodated within the electron-rich central cavity of the COF, forming stable host–guest assemblies through van der Waals interactions while preserving the structural integrity of the framework. Frontier molecular orbital and global reactivity analyses indicate that drug adsorption, particularly for IBU, significantly reduces the HOMO–LUMO gap and chemical hardness, enhancing electronic sensitivity, reactivity, and charge-transfer capability. Electron density difference and non-covalent interaction analyses confirm directional electron flow from the drugs to the COF and identify stabilizing interactions governing adsorption. Simulated UV–Vis spectra show pronounced red-shifts upon drug adsorption, with the IBU@TQBQ-COF complex exhibiting the largest effect. Thermodynamic analyses further confirm spontaneous, exothermic adsorption dominated by enthalpy contributions. Collectively, these findings highlight TQBQ-COF as a highly sensitive and robust platform for drug detection and removal in aqueous and biologically relevant environments.

**Supplementary Information:**

The online version contains supplementary material available at 10.1038/s41598-025-25834-3.

## Introduction

The growing presence of pharmaceutical contaminants in aquatic and terrestrial environments poses a significant threat to both ecosystems and human health^1–3^. Among these, widely used painkillers such as aspirin (ASP), paracetamol (PAR), and ibuprofen (IBU) are frequently detected in water bodies due to their extensive consumption and incomplete removal by conventional wastewater treatment processes^4–6^. These emerging contaminants are biologically active, often persistent, and capable of inducing toxicological effects even at trace concentrations. Consequently, there is an urgent need for the development of efficient and cost-effective adsorbent materials to capture and remove such pollutants from the environment.

A variety of materials, including carbon nanotubes (CNTs)^7^, metal–organic frameworks (MOFs)^8^, graphene-based materials^9^, and zeolites^10^, have been investigated for pharmaceutical adsorption. However, many of these materials suffer from drawbacks such as limited stability and high synthesis cost. CNTs often aggregate, which reduces their effective surface area, and they have limited chemical stability in aqueous solutions, along with high synthesis costs^11^. MOFs, while highly porous, can suffer from poor water stability, framework collapse, or leaching of metal ions, and require complex synthesis procedures^12^. Despite their high surface area, graphene-based materials generally exhibit low selectivity toward specific pharmaceutical compounds unless costly functionalization is applied^13^. Zeolites, although chemically stable and reusable, have relatively small pore sizes that limit the adsorption of larger molecules, and their surface chemistry often requires modification to enhance efficiency. In this regard, covalent organic frameworks (COFs) have emerged as a promising class of crystalline porous polymers composed of light elements (C, H, O, N, and B) linked through strong covalent bonds^15–17^. Owing to their high surface area, tunable pore size, structural regularity, and chemical robustness, COFs provide an excellent platform for capturing organic molecules, including pharmaceutical residues, with high efficiency.

Several experimental and computational studies have demonstrated the potential of COFs in molecular adsorption and sensing applications. Ghasemi et al.^18^, used molecular dynamics (MD) and density functional theory (DFT) to show that hydrogen bonding and van der Waals interactions dominate pharmaceutical adsorption, while pore-based COFs are less effective, confirming COFs’ efficiency and regenerability. Kaviani et al.^19^, showed that transition metal-doped oxo-triarylmethyl (TM@oxTAM), as a COF-inspired platform, strongly adsorbs ibuprofen with enhanced adsorption energies (− 1.20 to − 2.64 eV) and charge transfer through covalent and electrostatic interactions, highlighting its potential for removing pharmaceutical pollutants. Irshad et al.^20^, studied a thiazole-modified covalent triazine framework (S-CTF) for the adsorption of carcinogenic metabolites. They found that 2-amino-3-methylimidazo[4,5-f]quinoxaline (MEIQX) and 2-amino-1-methyl-6-phenylimidazo[4,5-b]pyridine (PhlP) exhibited the strongest interactions (− 24.58 kcal/mol), with efficient charge transfer. Zhuang et al.^21^, synthesized a TPB-DMTP covalent organic framework for sulfamerazine (SMT) removal from water. The COF exhibited high adsorption capacity (209 mg/g) and fast equilibrium (80 min), with SMT molecules primarily adsorbed via C–H··π interactions in the COF pores, as confirmed by DFT calculations.

There are a lot of COFs that have been developed for adsorption applications, including imine-, hydrazone-, and triazine-linked structures^22–24^. In 2020, Shi et al.^25^, designed a honeycomb-like covalent organic framework (TQBQ-COF) composed of triquinoxalinylene and benzoquinone units via the condensation reaction between tetraminophenone (TABQ) and cyclohexanehexaone (CHHO). This innovative TQBQ-COF contains multiple pyrazine and carbonyl groups, and both Fourier transform infrared (FTIR) spectroscopy and DFT calculations confirmed that the pyrazine (C = N) and carbonyl (C = O) moieties serve as the primary active sites of the framework. A DFT-based study by Mustafai et al.^26^, employed a theoretical approach to investigate the adsorption of toxic gases such as NH₃, HCN, H₂S, and PH₃ on TQBQ-COF, confirming its robustness and strong host–guest interactions, which further highlights its significance for adsorption and sensing applications. These unique structural and chemical features make TQBQ-COF highly promising candidates not only for environmental remediation but also for sensing of pharmaceutical and industrial pollutants in aqueous system.

**Fig. 1:**
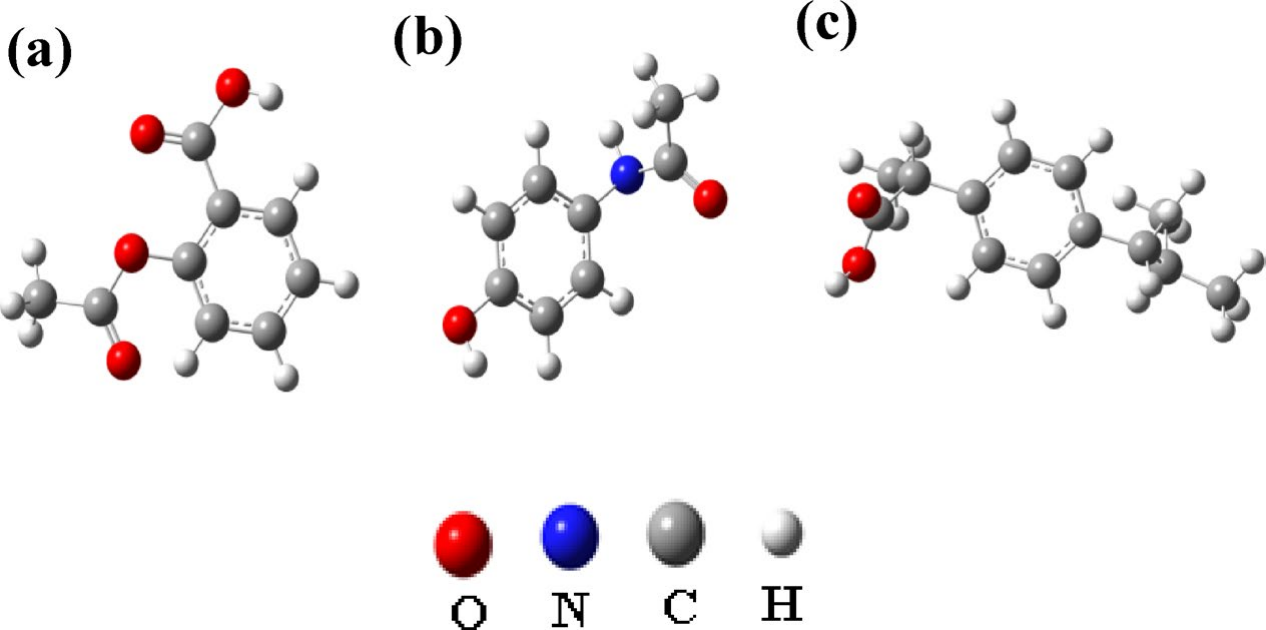
Chemical structure of (**a**) aspirin (ASP), (**b**) paracetamol (PCR), and (**c**) ibuprofen (IBU).

In this work, we employ first-principles DFT calculations to investigate the adsorption of aspirin, paracetamol, and ibuprofen molecules onto TQBQ-COF. The chemical structures of the considered drugs are shown in Fig. 1. The adsorption behavior is examined through interaction energy analysis, electronic structure evaluation, and charge-transfer characterization, alongside detailed studies using natural bond orbital (NBO), frontier molecular orbital (FMO), electron density difference (EDD), and non-covalent interaction (NCI), analyses. These insights provide a fundamental understanding of the interaction mechanisms, offering guidance for the rational design of COF-based adsorbents for pharmaceutical pollutant mitigation.

## Computational methods

Geometry optimization was carried out using the Gaussian 16 package^27^ at the B3LYP-D3/6-31G(d,p) level of theory, which has been widely reported as a reliable and computationally affordable level for predicting molecular geometries and energies of various adsorbent systems^28–32^. The inclusion of Grimme’s D3 dispersion correction has been widely reported to improve the reliability of calculations involving van der Waals (vdW) interactions^33^. All calculations were performed in water as the solvent using the polarizable continuum model (PCM)^34,35^, which efficiently captures the bulk polarization effects of the solvent by treating it as a continuous dielectric medium surrounding the solute. This approach provides a reliable description of solvent-induced stabilization of molecular orbitals and adsorption energies while maintaining computational efficiency^36^. Additionally, GaussView 6 was used to visualize and analyze the outputs from Gaussian calculations^37^. Vibrational frequency calculations confirmed that the optimized structures correspond to true minima on the potential energy surface. All systems were considered to be neutral, with a total charge of zero.

It is important to note that all computations in this study were performed using a molecular (cluster) approach rather than a fully periodic model. The COF was represented by a finite molecular fragment that captures the local adsorption environment. While this approach effectively describes local interactions and electronic structure changes upon drug interaction, it does not account for the long-range periodic effects inherent to extend the COF. Nonetheless, the molecular-level model provides valuable insights into the fundamental adsorption mechanisms and interaction characteristics that govern the overall interaction behavior of the COF.

The interaction energies of drug@TQBQ-COF systems were determined using the following expression:

1 $${\mathrm{E}}_{{{\mathrm{int}}}} = {\text{ E}}_{{{\mathrm{drug}}@{\mathrm{TQBQ}} - {\mathrm{COF}}}} {-} \, \left( {{\mathrm{E}}_{{{\mathrm{TQBQ}} - {\mathrm{COF}}}} + {\text{ E}}_{{{\mathrm{drug}}}} } \right)$$

Here, ${\mathrm{E}}_{{{\mathrm{drug}}@{\mathrm{TQBQ}} - {\mathrm{COF}}}}$ denotes the total energy of the complex, E_TQBQ-COF_ corresponds to the energy of the isolated TQBQ-COF, and E_drug_ refers to the energy of the free drug. The interaction energies were corrected for basis set superposition error (BSSE) using the counterpoise (CP) method^38–40^, as expressed in the following equation,

2 $${\mathrm{E}}_{{{\mathrm{int}},{\mathrm{cp}}}} = {\text{ E}}_{{{\mathrm{int}}}} {-}{\mathrm{BSSE}}$$

where E_int,cp_ represents the counterpoise-corrected interaction energy.

The thermodynamic stability of the systems was evaluated through the calculation of enthalpy change (ΔH), Gibbs free energy change (ΔG), and entropy change (ΔS), obtained from the following equations:

3 $$\Delta {\text{H }} = {\text{ H}}_{{{\mathrm{drug}}@{\mathrm{TQBQ}} - {\mathrm{COF}}}} {-}\left( {{\mathrm{H}}_{{{\mathrm{TQBQ}} - {\mathrm{COF}}}} + {\text{ H}}_{{{\mathrm{drug}}}} } \right)$$

4 $$\Delta {\text{G }} = {\text{ G}}_{{{\mathrm{drug}}@{\mathrm{TQBQ}} - {\mathrm{COF}}}} {-}\left( {{\mathrm{G}}_{{{\mathrm{TQBQ}} - {\mathrm{COF}}}} + {\text{ G}}_{{{\mathrm{drug}}}} } \right)$$

5 $$\Delta {\text{S }} = {\text{ S}}_{{{\mathrm{drug}}@{\mathrm{TQBQ}} - {\mathrm{COF}}}} {-}\left( {{\mathrm{S}}_{{{\mathrm{TQBQ}} - {\mathrm{COF}}}} + {\text{ S}}_{{{\mathrm{drug}}}} } \right)$$

Frontier molecular orbital (FMO) analysis was performed by calculating the energies of the highest occupied molecular orbital (E_HOMO_) and the lowest unoccupied molecular orbital (E_LUMO_), which allowed assessment of changes in the band gap. Additionally, Density of States (DOS) analysis was carried out using the GaussSum program^41^ to further investigate the electronic properties. Global reactivity descriptors derived from E_HOMO_ and E_LUMO_, including chemical hardness (η), chemical potential (μ), electrophilicity index (ω), and total charge transfer (ΔN), were calculated using the following expression^42^:

6 $$\eta = \frac{{{\mathrm{E}}_{{{\mathrm{LUMO}}}} - {\mathrm{E}}_{{{\mathrm{HOMO}}}} }}{2}$$

7 $$\mu = \frac{{{\mathrm{E}}_{{{\mathrm{LUMO}}}} + {\mathrm{E}}_{{{\mathrm{HOMO}}}} }}{2}$$

8 $$\omega = \frac{{\mu^{2} }}{2\eta }$$

9 $$\Delta N = \frac{{\left( {\mu_{TQBQ - COF } - \mu_{drug} } \right) }}{{2\left( {\eta_{TQBQ - COF } + \eta_{drug} } \right)}}$$

The mechanism, strength, and nature of the interactions were further investigated through Non-covalent Interaction (NCI), and electron density difference (EDD), analyses. NCI–reduced density gradient (RDG) calculations were carried out using Multiwfn^43^, and the resulting isosurfaces were visualized with Visual Molecular Dynamics (VMD)^44^. The interaction strength between the analytes and the surface was evaluated based on electron density and RDG values^45^.

Time-dependent density functional theory (TD-DFT) calculations were carried out at CAM-B3LYP/6-31G(d,p) to simulate the UV–Vis absorption spectra of the TQBQ-COF both before and after interaction with the drugs. The analysis considered the first 30 lowest-energy electronic excitations. This procedure enabled assessment of the changes in electronic excitation energies and spectral characteristics resulting from adsorption, providing insights into how interactions with the drugs alter the optical properties of the adsorbent.

## Results and discussion

### Optimized structures

**Fig. 2:**
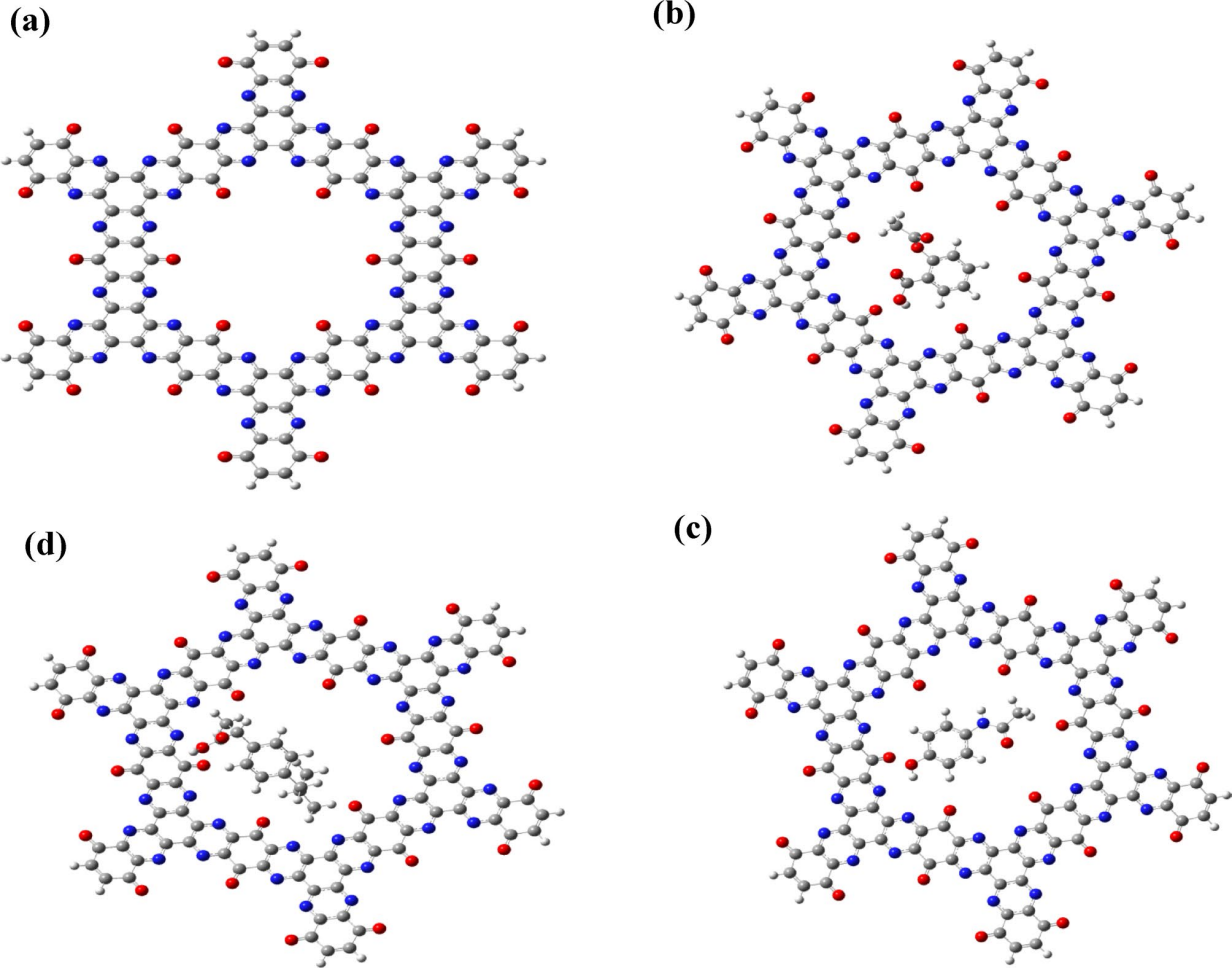
Optimized geometries of (**a**) pristine TQBQ-COF and its host–guest complexes with (**b**) aspirin (ASP), (**c**) paracetamol (PAR), and (**d**) ibuprofen (IBU).

The optimized geometry of TQBQ-COF is illustrated in Fig. 2a and is composed of multiple fused pyrazine (C = N), benzoquinone (C = O), and benzene rings. The twelve sp^2^-hybridized nitrogen atoms of the pyrazine rings and the six oxygen atoms of the benzoquinone rings are oriented toward the central cavity, creating an electron-rich region on the COF surface that serves as an effective binding site for drug molecules. In contrast, the carbon atoms of the aromatic rings show negligible charge accumulation. The electron-rich nitrogen and oxygen atoms within the cavity can interact strongly with electron-deficient sites of adsorbed analytes, leading to significant perturbations in the electronic structure of the COF.

The optimized geometries of TQBQ-COF and its complexes with the three drug molecules (ASP, PAR, and IBU) are shown in Fig. 2b–d, indicating that all drugs are accommodated within the central cavity of the COF. Upon adsorption, the drugs adopt stable orientations that maximize non-covalent interactions with the COF backbone. For clarity, lateral views of the drug@TQBQ-COF complexes are provided in the Supplementary Information (Fig. S1), showing the adsorption distances between the COF and the drug molecules. The measured distances are 2.65 Å (O_103_–H_198_), 2.67 Å (O_79_–H_195_), and 2.42 Å (O_158_–H_209_), with the strongest interaction observed for ibuprofen (IBU).

The COF framework maintains its overall structural integrity upon adsorption of the drug molecules, with no significant deviations observed. Among the studied drugs, IBU fits most snugly within the cavity, forming favorable interactions with the surrounding oxygen atoms of the COF, consistent with its stronger binding energy and greater electronic perturbation. These optimized structures highlight the ability of TQBQ-COF to effectively encapsulate drug molecules, providing a structural basis for enhanced electronic communication, efficient charge transfer, and improved sensing and adsorption capabilities.

### Electronic propertie

The FMO analysis was performed to examine the sensitivity, electron distribution, and reactivity of the TQBQ-COF toward drug molecule detection. A key parameter in this analysis is the HOMO–LUMO energy gap (E_g_), defined as the difference between the energies of the HOMO and the LUMO. Smaller variations in this gap can influence the electronic response of the material, affecting its sensitivity and conductivity. Consequently, a smaller HOMO–LUMO gap indicates higher sensitivity, enhanced reactivity, and improved conductivity. The percentage change in the energy gap (%ΔE_g_) upon interaction with target molecules is calculated using following equation:

10 $$\% \Delta {\mathrm{E}}_{{\mathrm{g}}} = \frac{{{\mathrm{E}}_{{\mathrm{g}}} \left( {{\mathrm{drug}}@{\mathrm{TQBQ}} - {\mathrm{COF}}} \right){ } - {\text{ E}}_{{\mathrm{g}}} \left( {{\mathrm{TQBQ}} - {\mathrm{COF}}} \right)}}{{{\mathrm{E}}_{{\mathrm{g}}} \left( {{\mathrm{TQBQ}} - {\mathrm{COF}}} \right)}} \times 100$$

where E_g_(drug@TQBQ-COF) and E_g_(TQBQ-COF) represent the energy gaps of the complex and pristine COF, respectively. As shown in Table 1, the isolated TQBQ-COF exhibits an energy gap of 2.41 eV, which is significantly lower than the 7.13 eV reported by Mustafai et al^26^. In their work, they performed all calculations in the gas phase, obtaining HOMO and LUMO energies of − 9.60 eV and − 2.47 eV, respectively. In contrast, the present study incorporates solvent effects (PCM–water), yielding HOMO and LUMO values of − 7.37 eV and − 4.96 eV, respectively. The inclusion of a polar solvent significantly stabilizes the unoccupied orbitals (particularly the LUMO) through polarization and charge redistribution, while slightly destabilizing the occupied orbitals, resulting in a narrower HOMO–LUMO gap. Therefore, the observed reduction in energy gap primarily arises from solvent polarization effects, rather than any methodological inconsistency, and is consistent with the expected electronic behavior of COF systems in aqueous environments. This pronounced reduction in the HOMO–LUMO gap arises from strong solvation and polarization effects, where water stabilizes the LUMO through polarization while slightly destabilizing the HOMO via orbital reorganization and charge redistribution. The combined effect of these interactions significantly enhances the electronic conductivity and reactivity of TQBQ-COF, making it more responsive for sensing and adsorption applications in aqueous environments. Upon drug adsorption, the HOMO–LUMO gap of all drug@TQBQ-COF complexes decreases relative to the pristine TQBQ-COF, reflecting an enhancement in electronic response and sensing performance. For ASP@TQBQ-COF, the HOMO shifts upward to − 6.08 eV while the LUMO remains unchanged at − 4.96 eV, resulting in a much narrower energy gap of 1.12 eV. Similarly, in PAR@TQBQ-COF, the HOMO increases to − 6.10 eV and the LUMO to − 5.00 eV, yielding energy gap of 1.10 eV. The most dramatic effect is observed in IBU@TQBQ-COF, where the HOMO rises significantly to − 5.45 eV, with the LUMO remaining at − 4.96 eV, reducing the gap to only 0.49 eV. Among the studied systems, the most pronounced reduction is observed for the IBU@TQBQ-COF complex, with a %ΔE_g_ of − 79.66% (Table 1). This considerable decrease demonstrates the strong electronic response of the COF toward IBU, arising from efficient charge transfer and strong binding affinity. Overall, these results confirm the outstanding sensing potential of TQBQ-COF, with the IBU complex standing out as the most effective candidate for drug detection and removal applications.

**Table 1:** HOMO energy (E_HOMO_), LUMO energy (E_LUMO_), energy gap (E_g_), and percentage change in energy gap (%ΔE_g_) for TQBQ-COF and drug@TQBQ-COF systems.

Compound	E_HOMO_ (eV)	E_LUMO_ (eV)	E_g_ (eV)	%ΔE_g_
TQBQ-COF	− 7.37	− 4.96	2.41	–
ASP@TQBQ-COF	− 6.08	− 4.96	1.12	− 53.25
PAR@ TQBQ-COF	− 6.10	− 5.00	1.10	− 54.35
IBU@ TQBQ-COF	− 5.45	− 4.96	0.49	− 79.66

**Fig. 3:**
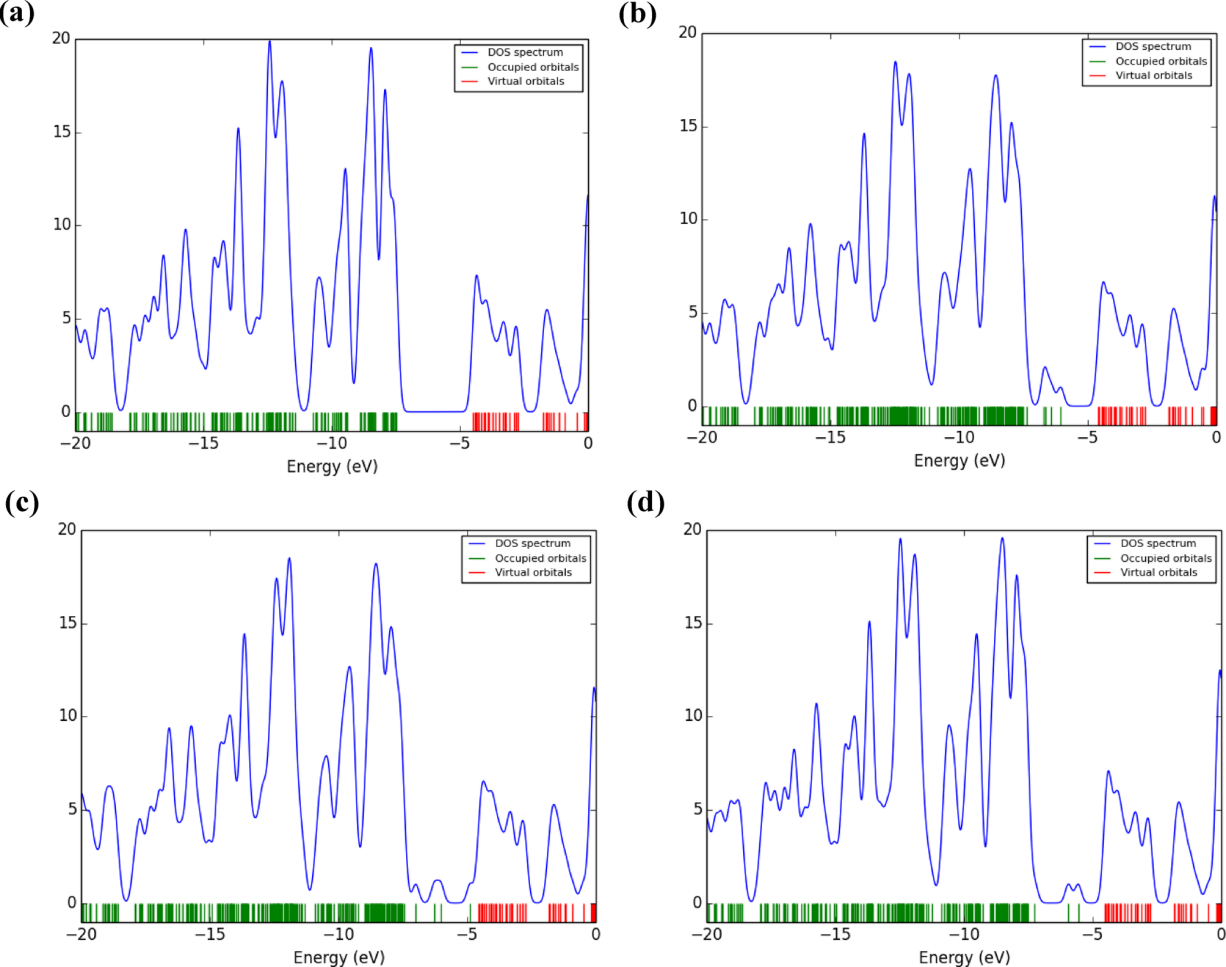
Graphical visualization of DOS spectra for (**a**) TQBQ-COF, (**b**) ASP@ TQBQ-COF, (**c**) PAR@ TQBQ-COF, and (**d**) IBU@ TQBQ-COF.

The density of states (DOS) analysis offers important insights into the electronic structure of TQBQ-COF and its interactions with various drug molecules, as illustrated in Fig. 3. For the pristine TQBQ-COF, the DOS profile displays a well-defined energy gap of 2.62 eV, reflecting moderate electronic conductivity and intrinsic stability. Upon adsorption of drug molecules, significant modifications occur in the DOS profiles of the resulting complexes, particularly near the Fermi level.

These changes indicate enhanced electronic interactions and charge redistribution between the TQBQ-COF and the adsorbed molecules. Specifically, drug adsorption leads to a shift of electronic states closer to the Fermi level, which facilitates electron mobility and increases conductivity. Among the studied drugs, IBU produces the most pronounced effect. The DOS shows a dramatic reduction in the energy gap, accompanied by a substantial increase in the density of states near the Fermi level. This observation signals strong electronic coupling and efficient charge transfer between IBU and TQBQ-COF. Overall, these DOS results corroborate the HOMO–LUMO analysis, confirming that TQBQ-COF exhibits high sensitivity toward drug adsorption, particularly for IBU. The pronounced electronic perturbations upon drug binding highlight the COF’s potential as a highly responsive platform for drug detection and removal, with enhanced conductivity facilitating rapid sensing and efficient charge transport.

### Global reactivity descriptors

Global reactivity descriptors, including chemical potential, chemical hardness, and electrophilicity, were employed to investigate how drug molecules influence the reactivity of TQBQ-COF. The reactivity indices of the isolated drug molecules are listed in Table S1, whereas those for TQBQ-COF and its drug-loaded complexes are summarized in Table 2. As shown in Table 2, adsorption of IBU onto TQBQ-COF leads to a notable decrease in chemical hardness, indicating that the resulting complex is softer and more chemically reactive. This softening enhances the flexibility of the electronic structure, enabling the COF to respond effectively to external stimuli such as drug adsorption.

**Table 2:** Chemical hardness (η), chemical potential (μ), electrophilicity (ω), and total amount of charge transfer (ΔN) for TQBQ-COF and drug@TQBQ-COF systems.

Compound	η (eV)	μ (eV)	ω (eV)	ΔN
TQBQ-COF	1.20	− 6.16	15.81	–
ASP@TQBQ-COF	0.56	− 5.52	27.20	− 0.21
PAR@ TQBQ-COF	0.55	− 5.55	28.00	− 0.34
IBU@ TQBQ-COF	0.24	− 5.20	56.33	− 0.32

Additionally, the chemical potential values of all complexes remain consistently negative, confirming their thermodynamic stability after interaction with the drugs and supporting their potential for practical sensing applications. Charge transfer (ΔN) analysis reveals negative values for all complexes, indicating that electrons are donated from the drug molecules to TQBQ-COF. This establishes the COF as an electron acceptor and the drugs as electron donors, highlighting strong donor–acceptor interactions and efficient charge redistribution during adsorption. Among the studied drugs, IBU exhibits the largest charge transfer and the greatest reduction in hardness, emphasizing its strong electronic coupling with the COF. Collectively, these results demonstrate that TQBQ-COF exhibits high sensitivity, enhanced chemical reactivity, and robust thermodynamic stability toward the adsorbed drug molecules. The combination of significant charge transfer, reduced hardness, and stable chemical potential positions TQBQ-COF as a promising platform for developing highly sensitive and reusable drug sensors. Furthermore, the pronounced electronic adaptability and strong donor–acceptor interactions indicate that this material could be highly effective for drug detection and removal under diverse environmental conditions, including aqueous and biologically relevant media.

### Adsorption energies and thermodynamic parameters

The adsorption energies and thermodynamic parameters for the drug@TQBQ-COF complexes are summarized in Table 3. In all cases, the calculated interaction energies (E_int_) are negative, clearly confirming that the adsorption of drug molecules onto the COF surface is energetically favorable in both the aqueous phases. This finding demonstrates that the interactions occur spontaneously and are thermodynamically viable under the studied conditions. Among the three investigated drug molecules, the IBU@TQBQ-COF complex emerges as the most stable, with adsorption energy of − 1.22 eV, which is consistent with its shortest interaction distance of 2.42 Å. This relatively large stabilization energy reflects a strong affinity between the COF framework and ibuprofen, which can be attributed to its favorable molecular size and geometry that allow optimal accommodation within the central cavity. Multiple non-covalent interactions contacts with the pore walls, synergistically contribute to the enhanced stabilization of this complex. The pronounced stability of IBU encapsulation highlights the potential of TQBQ-COF as an appropriate host for ibuprofen detection and sensing applications. To ensure accuracy, the interaction energies were corrected for basis set superposition error (BSSE) using the counterpoise method. The calculated BSSE values for all drug@TQBQ-COF complexes range from 0.003 to 0.078 eV, which are typical for weak interactions computed with the 6-31G(d,p) basis set^46,47^.

**Table 3:** Interaction energy (E_int_), basis set superposition error (BSSE), counterpoise corrected interaction energy (E_int,cp_), change in Gibbs free energy (ΔG), change in enthalpy (ΔH), and change in entropy (ΔS) for drug@TQBQ-COF complexes.

Complex	E_int_ (eV)	BSSE (eV)	E_int,cp_ (eV)	ΔH (eV)	ΔG (eV)	ΔS (eV/K)
ASP@TQBQ-COF	− 0.88	0.003	− 0.883	− 0.88	− 0.44	− 0.0014
PAR@ TQBQ-COF	− 0.91	0.078	− 0.988	− 0.90	− 0.57	− 0.0011
IBU@ TQBQ-COF	− 1.14	0.078	− 1.22	− 1.13	− 0.78	− 0.0012

Thermodynamic parameters further support these observations. The negative values of Gibbs free energy (ΔG) for all complexes indicate that adsorption is a spontaneous process, consistent with the exothermic nature revealed by the negative enthalpy changes (ΔH). These results suggest that the driving force for adsorption arises primarily from enthalpy contributions, associated with strong host–guest interactions within the COF cavity. On the other hand, the entropy changes (ΔS) were consistently negative, which reflects the loss of degrees of freedom upon complex formation. This reduction in entropy is expected because free drug molecules in solution possess higher translational and rotational freedom compared to when confined within the rigid COF cavity. Interestingly, the magnitude of the enthalpy change is greater than that of the Gibbs free energy, indicating that enthalpy stabilization outweighs the unfavorable entropic contributions. Such a balance is typical for adsorption phenomena in porous frameworks, where the gain in interaction energy compensates for the ordering effect introduced by confinement.

### Electron density difference analysis

**Fig. 4:**
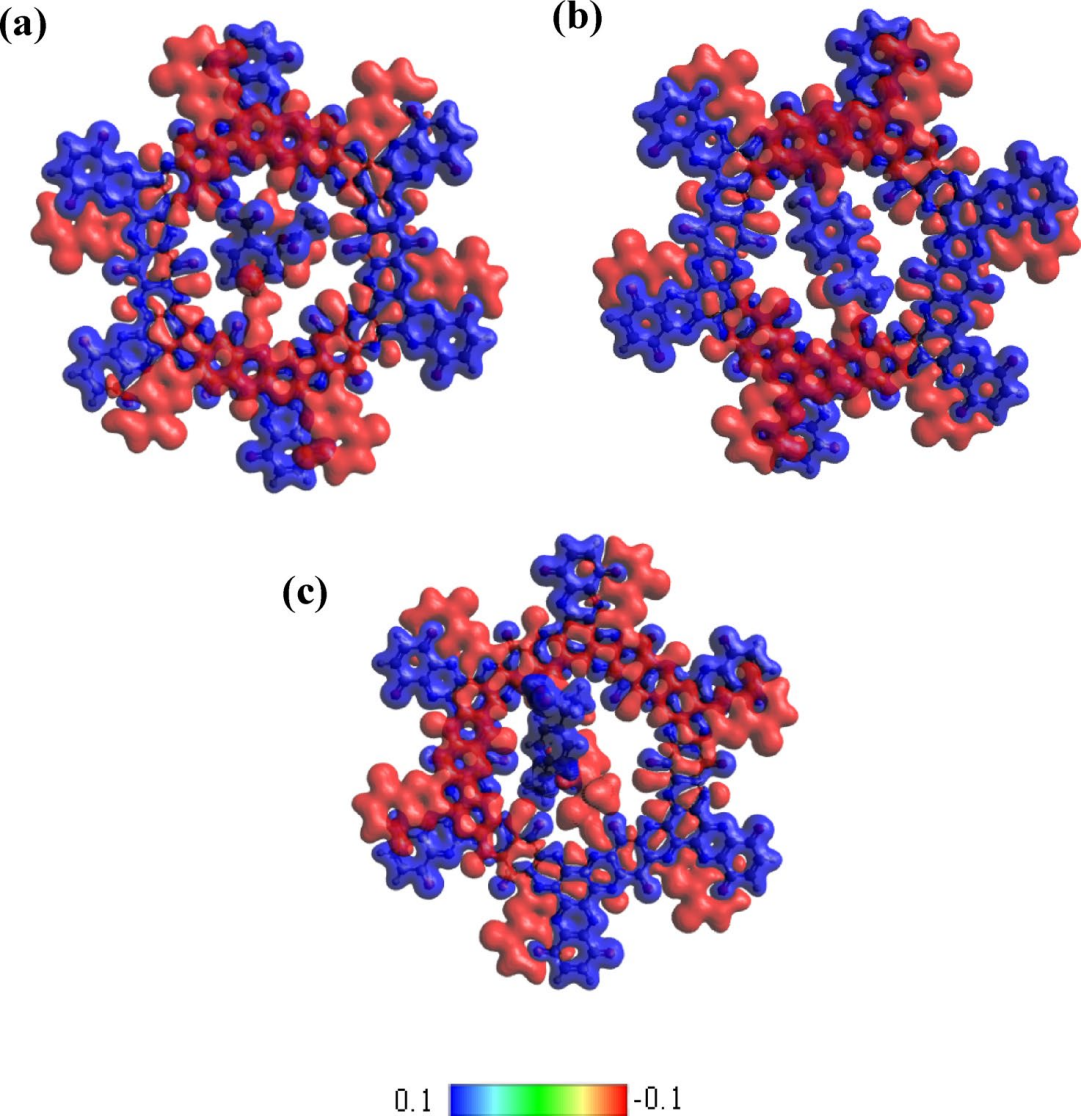
Electron density difference (EDD) maps of (**a**) ASP@TQBQ-COF, (**b**) PAR@TQBQ-COF, and (**c**) IBU@TQBQ-COF.

The electron density difference (EDD) analysis provides a powerful visualization of how electronic charge redistributes when drug molecules interact with the TQBQ-COF framework. By comparing the total electron density of the drug–COF complex with the sum of the densities of the isolated components, detailed EDD maps can be generated (Fig. 4). These maps reveal distinct regions of charge redistribution, where red isosurfaces correspond to areas of electron accumulation and blue isosurfaces indicate electron depletion. Notably, the drug molecules are predominantly surrounded by blue regions, signifying that they act as electron donors, while the TQBQ-COF serves as an electron acceptor. This directional charge transfer highlights the strong electronic coupling between the adsorbates and the framework. Such behavior is consistent with the results of charge transfer calculations, where negative charge is observed to flow preferentially from the drug molecules toward the COF backbone. The simultaneous presence of electron-rich and electron-deficient regions suggests that orbital overlap and donor–acceptor interactions are central to stabilizing the complexes.

Overall, the EDD findings complement the energetic, electronic, and topological analyses, providing strong evidence for charge transfer as the dominant interaction mechanism. These insights not only validate the role of TQBQ-COF as a highly responsive material for detecting drug molecules but also emphasize its potential application in removal technologies, where efficient electron exchange plays a critical role in sensing performance.

### QTAIM analysis

In the QTAIM analysis, several critical parameters were examined at the bond critical points (BCPs) to elucidate the nature of the intermolecular interactions. These included the electron density (ρ(r)), Laplacian of electron density (∇^2^ρ(r)), kinetic energy density (G(r)), potential energy density (V(r)), and total energy density (H(r)). The identified BCPs are depicted in the topological graphs in Fig. S2, and their corresponding parameters are summarized in Table S2.

The strength of the interactions can be inferred from the ρ(r) values. Typically, ρ(r) values above 0.1 a.u. indicate strong covalent bonds, while values below 0.1 a.u. correspond to weaker, non-covalent interactions^36^. As presented in Table S2, all complexes exhibit ρ(r) values in the range of 0.007–0.094 a.u., signifying the dominance of weak non-covalent interactions. Furthermore, the positive values of both ∇^2^ρ(r) and H(r) across all complexes confirm the presence of closed-shell (non-covalent) interactions. The ratio of –G(r)/V(r) provides additional insight into the bonding nature. According to the classification by Espinosa et al.^48^, a ratio greater than 1 denotes purely non-covalent interactions, values between 0.5 and 1 suggest electrostatic interactions with partial covalent character, and ratios below 0.5 correspond to covalent bonds. In all drug@ TQBQ-COF complexes, the –G(r)/V(r) ratios were consistently above 1, unequivocally confirming the non-covalent character of the adsorption. Overall, the QTAIM results align with the electronic structure and energy analyses, indicating that the interaction between the drug molecules and the TQBQ-COF framework is primarily governed by weak, non-covalent forces.

### NCI analysis

**Fig. 5:**
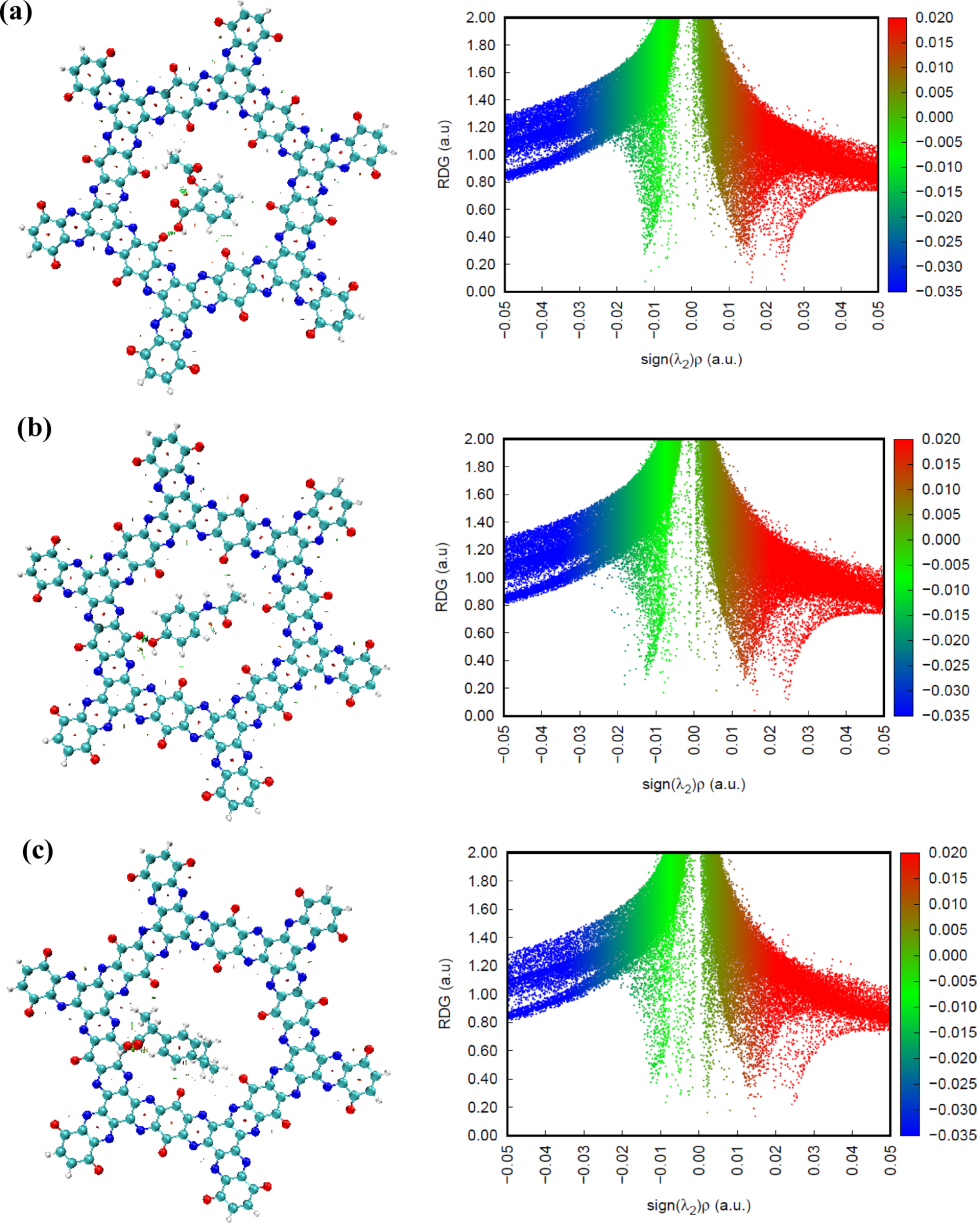

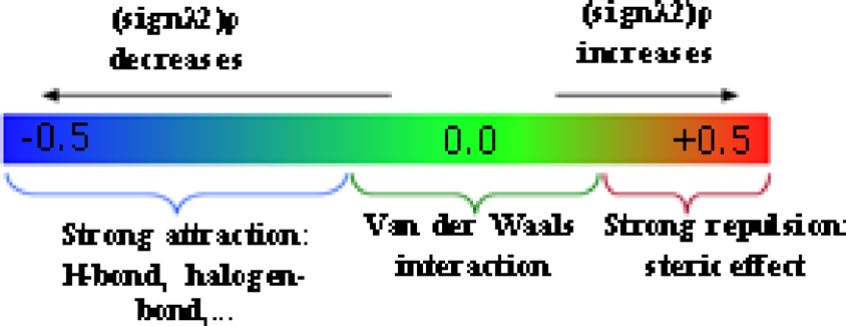
3D isosurface map (left side) reduced density gradient (RDG) graph (right side) and for (**a**) ASP@TQBQ-COF, (**b**) PAR@ TQBQ-COF, and (**c**) IBU@TQBQ-COF.

Non-covalent interaction (NCI) analysis was performed to probe the closed-shell interactions governing the adsorption of drug molecules within the TQBQ-COF framework. The weak interactions were evaluated through reduced density gradient (RDG) analysis, combining 2D scatter plots with their corresponding 3D iso-surface visualizations, as presented in Fig. 5. In the RDG scatter maps, the sign of λ₂(r)ρ(r) serves as a descriptor of interaction type: blue regions (λ₂(r)ρ(r) < 0) indicate strong attractive forces such as hydrogen bonding; red regions (λ₂(r)ρ(r) > 0) correspond to steric clashes or repulsive interactions; and green regions (λ₂(r)ρ(r) ≈ 0) represent weak van der Waals interactions. For all the drug@TQBQ-COF complexes, the 2D RDG plots clearly exhibit pronounced green domains near λ₂(r)ρ(r) ≈ 0, confirming that van der Waals interactions dominate the adsorption process. These green regions correspond well to the extended green iso-surfaces in the 3D NCI visualizations, which are mainly located between the encapsulated drug molecules and the inner cavity walls of the COF framework. This correspondence between the 2D and 3D representations highlights the consistency and reliability of the observed non-covalent interactions.

Meanwhile, small red spikes in the RDG plots, localized within the λ₂ range of 0.01–0.05 a.u., correspond to red patches on the 3D iso-surfaces. These features are primarily situated within the aromatic regions of the TQBQ-COF, reflecting steric hindrance and repulsive effects inherent to the dense π-electron system of the benzene and pyrazine rings. Although these repulsive contributions exist, they are relatively minor compared to the stabilizing effects of the van der Waals forces that dominate the overall host–guest interaction.

### UV–Vis analysis

**Fig. 6:**
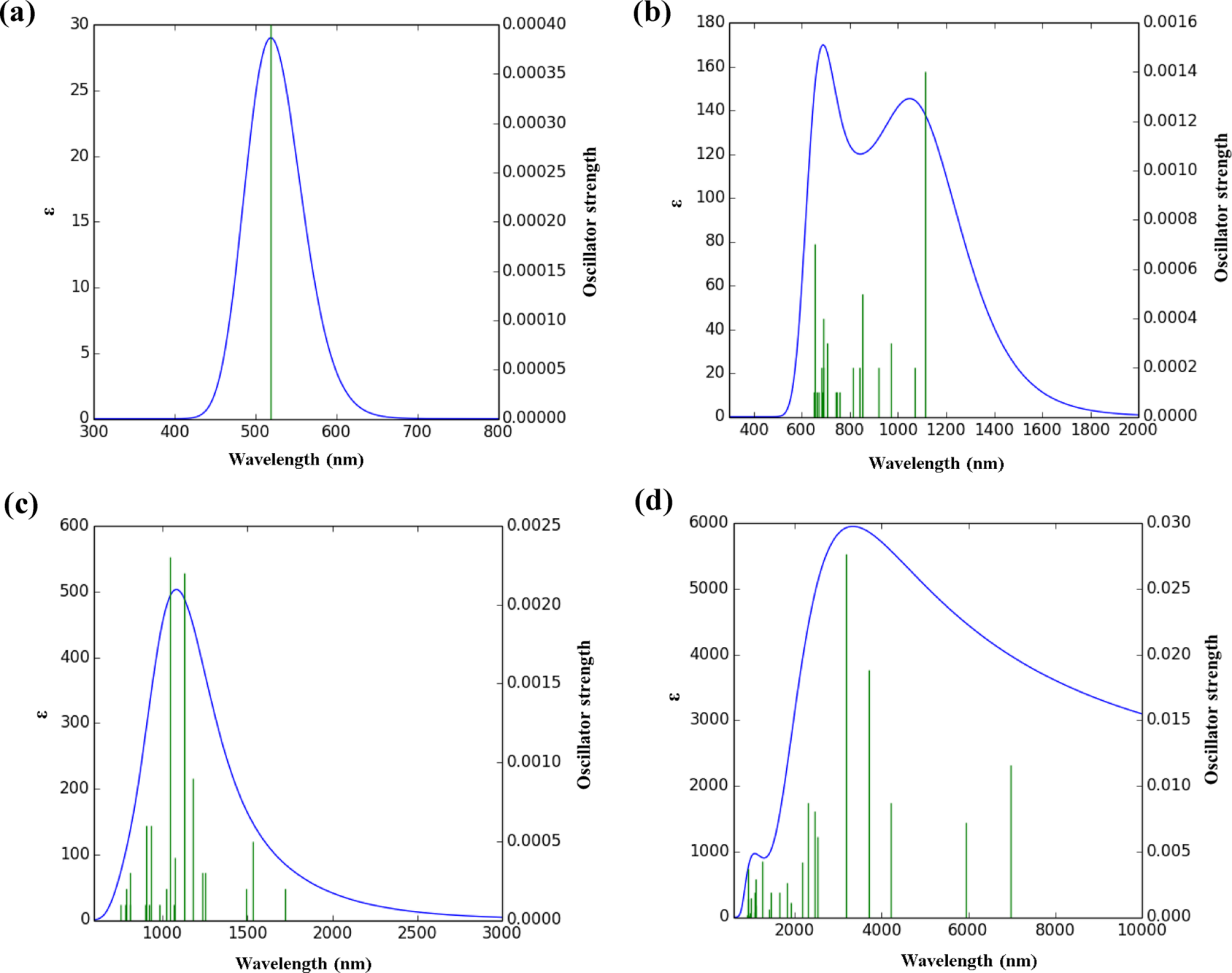
Graphical illustration of UV–Vis spectra for (**a**) TQBQ-COF, (**b**) ASP@TQBQ-COF, (**c**) PAR@ TQBQ-COF, and (**d**) IBU@ TQBQ-COF.

To better understand the interaction mechanisms between drug molecules and the TQBQ-COF, we analyzed the UV–Vis absorption spectra of the pristine TQBQ-COF and its drug-adsorbed complexes (Fig. 6). This analysis provides insight into the optical and electronic modifications induced by drug adsorption. The main spectral parameters examined were the maximum absorption wavelength (λ_max_), excitation energy (E_exc_), and oscillator strength (f), as summarized in Table 4. The pristine TQBQ-COF displays a dominant absorption peak at 518.76 nm, corresponding to excitation energy of 2.39 eV and oscillator strength of 0.0004, characteristic of its electronic transitions. After drug adsorption, clear spectral variations emerge, most notably red-shifts in the absorption peaks, which indicate lowered excitation energies and altered electronic structures. These shifts reflect enhanced charge-transfer interactions and narrowing of the HOMO–LUMO gap in the complexes. Among all systems, the IBU@TQBQ-COF complex exhibits the largest red-shift, with λ_max_ shifting to 3173.38 nm and E_exc_ decreasing to 0.39 eV. This significant change highlights a strong interaction between IBU and the TQBQ-COF, likely driven by pronounced charge transfer and orbital overlap. The most intense excitation is attributed to the HOMO → L + 7 transition with a dominant contribution of 92%, further confirming the strong electronic coupling between IBU and the framework. Overall, the results confirm that the optical response of the TQBQ-COF is highly sensitive to drug adsorption, with IBU producing the most distinct effect. It should be emphasized that the observed red-shifts should be interpreted qualitatively, providing insight into relative interactions rather than absolute excitation energies^49,50^.

**Table 4:** Maximum absorption wavelength (λ_max_), excitation energy (E_exc_), oscillation strength (f), and main transitions for TQBQ-COF and drug@TQBQ-COF complexes.

Compound	λ_max_ (nm)	E_exc_ (eV)	f	Main transitions
TQBQ-COF	518.76	2.39	0.0004	H-17 → L + 8 (13%)
				H-16 → L + 6 (11%)
				H-15 → L + 7 (11%)
ASP@ TQBQ-COF	1114.66	1.11	0.0014	HOMO → LUMO (81%)
				HOMO → L + 1 (15%)
PAR@ TQBQ-COF	1815.55	0.68	0.0026	HOMO → L + 13 (85%)
				HOMO → L + 12 (3%)
IBU@ TQBQ-COF	3173.38	0.39	0.027	HOMO → L + 7 (92%)
				HOMO → LUMO (3%)

## Conclusion

In this study, we systematically investigated the structural, electronic, and optical properties of triquinoxalinylene and benzoquinone-based covalent organic framework (TQBQ-COF) and its interactions with three drug molecules, including aspirin (ASP), paracetamol (PAR), and ibuprofen (IBU), using a DFT-based computational approach. The optimized geometries reveal that the drugs are efficiently accommodated within the electron-rich central cavity of the COF, forming stable host–guest assemblies through van der Waals interactions, while preserving the overall framework integrity. Frontier molecular orbital and global reactivity analyses indicate significant reductions in the HOMO–LUMO gap and chemical hardness, particularly for the IBU@TQBQ-COF complex, reflecting enhanced electronic sensitivity, reactivity, and efficient charge transfer. Electron density difference and non-covalent interaction analyses confirm directional electron flow from the drug molecules to the COF and highlight the key stabilizing interactions that govern adsorption. UV–Vis spectral simulations reveal pronounced red-shifts upon drug adsorption, with IBU inducing the largest effect. Thermodynamic analyses further support spontaneous, exothermic adsorption driven predominantly by enthalpy contributions. Collectively, these findings establish TQBQ-COF as a highly sensitive and robust platform for drug detection and removal, with potential applications in aqueous and biologically relevant environments.

## Supplementary Information

Below is the link to the electronic supplementary material.

Supplementary Material 1

## Data Availability

Data sets generated during the current study are available from the corresponding author (Sami Bawazeer) on reasonable request.
