# Supplementary material for: First-principles investigation of aspirin, paracetamol, and ibuprofen adsorption on triquinoxalinylene and benzoquinone-based covalent organic framework
Source: Sci Rep. 2026 Apr 10;16:16162. doi: 10.1038/s41598-025-25834-3 (PMC13201824; doi:10.1038/s41598-025-25834-3)
Supplement: Supplementary file 1 — Supplementary Material 1 [file 41598_2025_25834_MOESM1_ESM.docx]

**First-Principles Investigation of Aspirin, Paracetamol, and Ibuprofen Adsorption on Triquinoxalinylene and Benzoquinone-Based Covalent Organic Framework**

Sami Bawazeer

Department of Pharmaceutical Sciences, Faculty of Pharmacy, Umm Al-Qura

University, Makkah, Saudi Arabia

[Sami.Bawww@proton.me](mailto:Sami.Bawww@proton.me)

**Encapsulation of Drug Molecules in COF Cavities**

For each adsorption complex, the drug molecule (aspirin, paracetamol, or ibuprofen) was initially positioned within the central cavity of the TQBQ-COF fragment to model the encapsulation process. This placement was chosen to maximize potential interactions with the COF framework, including van der Waals contacts. After initial placement, full geometry optimization was performed using the **B3LYP-D3/6-31G(d,p)** level of theory in water (PCM) to allow the system to relax to the most stable configuration. This approach ensures that the drug molecule is properly accommodated in the COF cavity while capturing realistic interaction patterns representative of encapsulation processes.

**Fig. S1**. Views of the optimized drug@TQBQ-COF complexes showing the interaction distances for (a) aspirin (ASP), (b) paracetamol (PAR), and (c) ibuprofen (IBU).

| **Table S1.**  Energy of HOMO (E_HOMO_), energy of LUMO (E_LUMO_), energy gap (E_g_), chemical hardness (η), and chemical potential (μ) of drug molecules. | | | | | |
| --- | --- | --- | --- | --- | --- |
| **Compound** | **E_HOMO_ (eV)** | **E_LUMO_ (eV)** | **E_g_ (eV)** | **η (eV)** | **μ (eV)** |
| ASP | -7.15 | -1.75 | 5.40 | 2.70 | -4.45 |
| PAR | -6.18 | -0.91 | 5.27 | 2.63 | -3.54 |
| IBU | -6.45 | -0.50 | 5.95 | 2.97 | -3.47 |
|  |  |  |  |  |  |


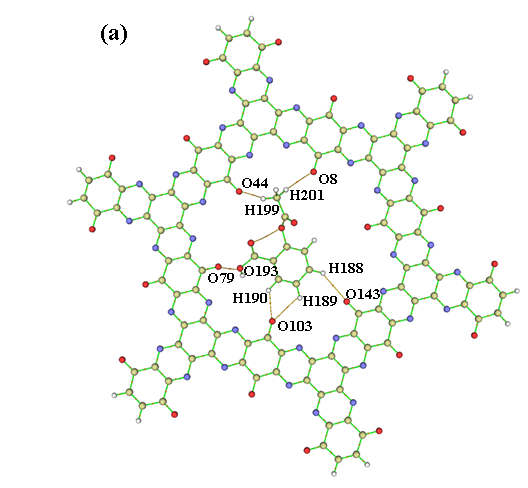


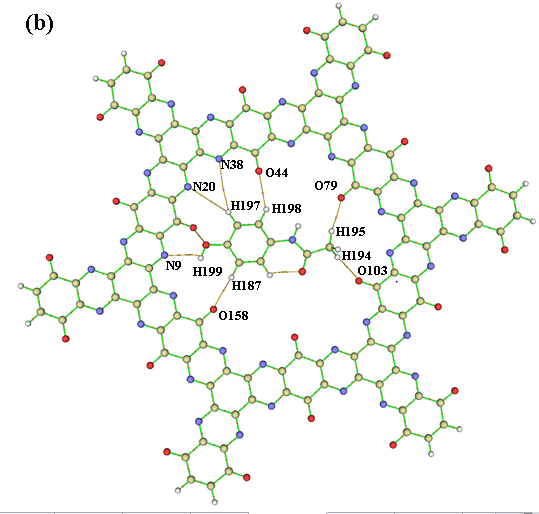


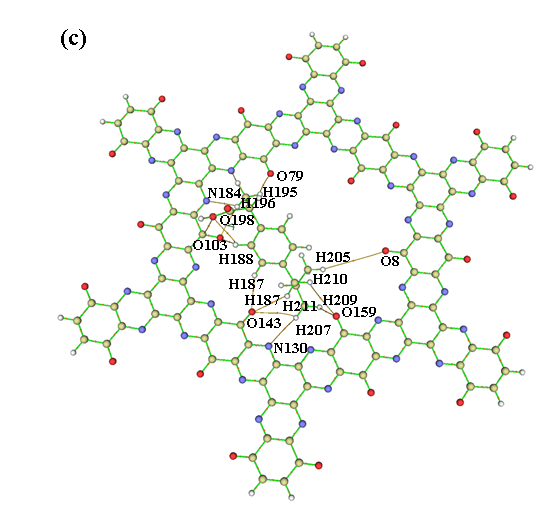


**Figure S2**. Quantum theory of atoms in molecule (QTAIM) molecular graphs for (a) ASP@TQBQ-COF, (b) PAR@TQBQ-COF, and (c)IBU@TQBQ-COF.

| **Table S2.**  Topological parameters (in a.u.) at BCP for intermolecular interactions in drug@TQBQ-COF complexes. | | | | | | | |
| --- | --- | --- | --- | --- | --- | --- | --- |
| **Complex** | **Bond** | **ρ(r)** | $\boldsymbol{\nabla}^{\mathbf{2}}\boldsymbol{\rho}$**(r)** | **H(r)** | **G(r)** | **V(r)** | **-G(r)/V(r)** |
| ASP@TQBQ-COF | H199$-$O144  H201$-$O8  H188$-$O143  H189$-$O103  H190$-$O103  H193$-$O79 | 0.070  0.066  0.045  0.065  0.089  0.011 | 0.271  0.253  0.189  0.274  0.365  0.454 | 0.011  0.010  0.010  0.013  0.015  0.012 | 0.056  0.051  0.037  0.055  0.076  0.050 | -0.045  -0.041  -0.027  -0.042  -0.061  -0.038 | 1.24  1.24  1.37  1.31  1.24  1.31 |
| PAR@TQBQ-COF | H198$-$O144  H197$-$N38  H197$-$N20  H199$-$N9  H187$-$O158  H188$-$O192  H195$-$O79  H194$-$O103  H195$-$O79 | 0.060  0.007  0.012  0.081  0.071  0.024  0.094  0.024  0.094 | 0.209  0.031  0.047  0.328  0.275  0.169  0.347  0.171  0.347 | 0.011  0.001  0.003  0.014  0.011  0.184  0.011  0.204  0.011 | 0.041  0.005  0.007  0.068  0.057  0.673  0.075  0.683  0.075 | -0.030  -0.004  -0.004  -0.054  -0.046  -0.489  -0.064  -0.479  -0.064 | 1.36  1.25  1.75  1.25  1.24  1.37  1.17  1.42  1.17 |
| IBU@ TQBQ-COF | O79$-$H195  O103$-$H196  O198$-$H188  N74$-$H194  N84$-$H196  H189$-$O103  H205$-$O8  H187$-$O143  H211$-$O143  H207$-$O143  H209$-$O159  H210$-$O159 | 0.049  0.051  0.088  0.025  0.056  0.075  0.025  0.087  0.022  0.034  0.036  0.016 | 0.194  0.202  0.332  0.092  0.198  0.291  0.254  0.334  0.100  0.146  0.149  0.061 | 0.010  0.009  0.012  0.005  0.010  0.011  0.014  0.012  0.007  0.009  0.008  0.004 | 0.038  0.040  0.071  0.017  0.039  0.060  0.039  0.071  0.018  0.028  0.028  0.010 | -0.028  -0.031  -0.059  -0.012  -0.029  -0.049  -0.015  -0.059  0.012  0.019  0.020  0.006 | 1.35  1.30  1.20  1.41  1.34  1.22  1.62  1.20  1.50  1.47  1.40  1.66 |

**Cartesian coordinates (Angstrom):**

**TQBQ-COF**

**X Y Z**

C -8.41682000 -3.40070000 0.25484000

C -7.19757000 -2.69768000 0.28318000

C -5.87257000 -3.41086000 0.36411000

C -5.91085000 -4.91071000 0.20828000

C -7.12957000 -5.61375000 0.13665000

C -8.46321000 -4.90847000 0.21305000

O -9.50134000 -5.51554000 0.22972000

O -4.84250000 -2.81806000 0.56187000

N -4.74333000 -5.53409000 0.15808000

C -4.77193000 -6.85989000 0.02540000

C -5.99358000 -7.56443000 -0.05428000

N -7.16438000 -6.93111000 0.00774000

C -3.49896000 -7.59123000 -0.03567000

C -3.49954000 -8.99819000 -0.16343000

C -4.77091000 -9.72849000 -0.25819000

C -5.99244000 -9.02565000 -0.20954000

N -9.58122000 -2.77042000 0.25105000

C -9.54731000 -1.43838000 0.26677000

C -8.32307000 -0.73316000 0.26351000

N -7.15640000 -1.37478000 0.26926000

C -10.81958000 -0.70382000 0.27853000

C -10.81944000 0.70641000 0.27835000

C -9.54702000 1.44070000 0.26649000

C -8.32293000 0.73521000 0.26350000

N -11.95824000 -1.40034000 0.28819000

C -13.08187000 -0.70326000 0.29854000

C -13.08173000 0.70632000 0.29836000

N -11.95795000 1.40317000 0.28783000

C -14.38186000 -1.47361000 0.30933000

C -15.62865000 -0.66724000 0.31943000

C -15.62852000 0.67082000 0.31926000

C -14.38157000 1.47694000 0.30896000

O -14.40559000 2.68604000 0.30836000

O -14.40614000 -2.68270000 0.30903000

N -9.58065000 2.77276000 0.25054000

C -8.41612000 3.40280000 0.25442000

C -7.19703000 2.69953000 0.28323000

N -7.15614000 1.37663000 0.26955000

C -8.46213000 4.91056000 0.21231000

C -7.12828000 5.61551000 0.13631000

C -5.90974000 4.91221000 0.20843000

C -5.87187000 3.41238000 0.36450000

O -9.50012000 5.51789000 0.22851000

O -4.84207000 2.81928000 0.56269000

N -7.16275000 6.93286000 0.00723000

C -5.99178000 7.56590000 -0.05454000

C -4.77032000 6.86109000 0.02551000

N -4.74206000 5.53531000 0.15842000

C -5.99028000 9.02711000 -0.20994000

C -4.76858000 9.72967000 -0.25842000

C -3.49740000 8.99907000 -0.16338000

C -3.49716000 7.59213000 -0.03545000

N -7.16222000 9.65888000 -0.30694000

C -7.11909000 10.97291000 -0.44839000

C -5.89806000 11.67584000 -0.49069000

N -4.73328000 11.05702000 -0.39666000

C -8.43482000 11.70655000 -0.57046000

C -8.35862000 13.17984000 -0.73851000

C -7.19961000 13.84729000 -0.77594000

C -5.88012000 13.17858000 -0.65138000

O -9.49407000 11.12417000 -0.53588000

O -4.84512000 13.80342000 -0.67923000

N -2.35911000 9.69027000 -0.20522000

C -1.23182000 8.99900000 -0.13147000

C -1.23149000 7.59472000 -0.01976000

N -2.35947000 6.90205000 0.03112000

C 0.04964000 9.79656000 -0.17142000

C 1.33212000 9.00168000 -0.11945000

C 1.33264000 7.59683000 -0.01463000

C 0.05102000 6.80029000 0.04315000

N 2.45906000 9.69525000 -0.17693000

C 3.59794000 9.00516000 -0.13030000

C 3.59819000 7.59700000 -0.01992000

N 2.46105000 6.90508000 0.03569000

C 4.86966000 9.73788000 -0.20071000

C 6.09192000 9.03580000 -0.14882000

C 6.09333000 7.57178000 -0.02053000

C 4.87128000 6.86572000 0.03420000

O 0.05171000 5.60091000 0.13140000

O 0.04852000 10.99736000 -0.24076000

N 7.26328000 6.93655000 0.03874000

C 7.22647000 5.61633000 0.13965000

C 6.00749000 4.91171000 0.18032000

N 4.84117000 5.53749000 0.13275000

N 4.83433000 11.06704000 -0.31905000

C 5.99928000 11.68876000 -0.38919000

C 7.22065000 10.98697000 -0.34102000

N 7.26396000 9.67082000 -0.21994000

C 5.98072000 13.19355000 -0.52877000

C 7.30041000 13.86598000 -0.62844000

C 8.45994000 13.19990000 -0.58403000

C 8.53649000 11.72479000 -0.43261000

C 8.55820000 4.90835000 0.21291000

C 8.51089000 3.39942000 0.25465000

C 7.29127000 2.69481000 0.27589000

C 5.95971000 3.40590000 0.27812000

N 9.67726000 2.77167000 0.26649000

C 9.64716000 1.43948000 0.28807000

C 8.42398000 0.73361000 0.29599000

N 7.25424000 1.37111000 0.29582000

O 4.94510000 13.81724000 -0.56000000

O 9.59644000 11.14431000 -0.38759000

O 4.91953000 2.80660000 0.34984000

O 9.59823000 5.51228000 0.23304000

C 10.91936000 0.70417000 0.29834000

C 10.91919000 -0.70674000 0.29831000

C 9.64682000 -1.44176000 0.28801000

C 8.42380000 -0.73560000 0.29595000

N 9.67660000 -2.77395000 0.26637000

C 8.51008000 -3.40142000 0.25449000

C 7.29063000 -2.69653000 0.27573000

N 7.25391000 -1.37282000 0.29572000

N 12.05824000 1.40033000 0.30543000

C 13.18194000 0.70322000 0.31281000

C 13.18178000 -0.70633000 0.31278000

N 12.05791000 -1.40318000 0.30537000

C 14.48206000 1.47358000 0.32107000

C 15.72882000 0.66719000 0.32595000

C 15.72866000 -0.67091000 0.32593000

C 14.48171000 -1.47700000 0.32100000

C 8.55704000 -4.91037000 0.21273000

C 7.22514000 -5.61803000 0.13942000

C 6.00633000 -4.91311000 0.18003000

C 5.95890000 -3.40730000 0.27787000

O 14.50595000 2.68264000 0.32354000

O 14.50532000 -2.68607000 0.32342000

N 7.26163000 -6.93827000 0.03852000

C 6.09154000 -7.57321000 -0.02078000

C 4.86966000 -6.86685000 0.03386000

N 4.83986000 -5.53862000 0.13240000

C 6.08978000 -9.03723000 -0.14903000

C 4.86735000 -9.73901000 -0.20095000

C 3.59580000 -9.00598000 -0.13064000

C 3.59639000 -7.59783000 -0.02033000

N 7.26166000 -9.67254000 -0.22005000

C 7.21804000 -10.98869000 -0.34108000

C 5.99650000 -11.69018000 -0.38929000

N 4.83170000 -11.06818000 -0.31924000

C 8.53370000 -11.72684000 -0.43255000

C 8.45679000 -13.20193000 -0.58393000

C 7.29711000 -13.86773000 -0.62838000

C 5.97758000 -13.19498000 -0.52881000

O 4.91886000 -2.80775000 0.34954000

O 9.59693000 -5.51454000 0.23285000

O 4.94181000 -13.81841000 -0.56006000

O 9.59379000 -11.14662000 -0.38750000

N -2.36109000 -6.90141000 0.03069000

C -1.23328000 -7.59436000 -0.02024000

C -1.23396000 -8.99865000 -0.13181000

N -2.36142000 -9.68966000 -0.20535000

C 0.04942000 -6.80023000 0.04250000

C 1.33084000 -7.59709000 -0.01517000

C 1.32998000 -9.00195000 -0.11988000

C 0.04731000 -9.79651000 -0.17184000

N 2.45942000 -6.90561000 0.03518000

N 2.45675000 -9.69580000 -0.17728000

O 0.04590000 -10.99731000 -0.24122000

O 0.05041000 -5.60083000 0.13049000

N -4.73594000 -11.05586000 -0.39630000

C -5.90089000 -11.67443000 -0.49007000

C -7.12175000 -10.97121000 -0.44762000

N -7.16455000 -9.65716000 -0.30628000

C -5.88331000 -13.17718000 -0.65061000

C -7.20299000 -13.84561000 -0.77490000

C -8.36183000 -13.17788000 -0.73734000

C -8.43766000 -11.70456000 -0.56942000

O -9.49677000 -11.12194000 -0.53470000

O -4.84846000 -13.80227000 -0.67858000

H 9.41736000 13.70541000 -0.65716000

H 7.25995000 14.94474000 -0.73952000

H 9.41409000 -13.70768000 -0.65699000

H 7.25638000 -14.94648000 -0.73943000

H -7.16309000 -14.92301000 -0.89866000

H -9.31910000 -13.68059000 -0.82931000

H -16.54951000 -1.24154000 0.32702000

H -16.54926000 1.24531000 0.32671000

H -9.31576000 13.68276000 -0.83069000

H -7.15946000 14.92468000 -0.89979000

H 16.64972000 1.24143000 0.33019000

H 16.64943000 -1.24536000 0.33013000

**ASP@TQBQ-COF**

C -8.83260887 -2.56771435 0.25251753

C -7.61921102 -1.86676115 0.25355077

C -6.28548323 -2.63536558 0.22464314

C -6.28524820 -4.17423781 0.17679684

C -7.49868727 -4.87493055 0.15646279

C -8.83248722 -4.10681429 0.20310393

O -9.92193190 -4.73663305 0.19950322

O -5.19594872 -2.00571698 0.23906280

N -5.12680350 -4.84216302 0.15257132

C -5.12683993 -6.17795778 0.08539996

C -6.34033156 -6.87740703 0.04771539

N -7.49875660 -6.21077198 0.09545383

C -3.79313650 -6.94716589 0.04803874

C -3.79302577 -8.34576929 -0.04059607

C -5.12787464 -9.11136257 -0.09879523

C -6.33813569 -8.41388918 -0.05109561

N -9.99091534 -1.89990653 0.29339934

C -9.99064238 -0.56236373 0.31333470

C -8.77747248 0.13836427 0.29644671

N -7.61927898 -0.52976912 0.27835660

C -11.32231754 0.20971255 0.35303454

C -11.32213021 1.60759805 0.35318003

C -9.99026781 2.37933270 0.31338600

C -8.77727707 1.67830341 0.29653575

N -12.48384208 -0.44960720 0.38740693

C -13.59900996 0.20793472 0.42876096

C -13.59881422 1.60999941 0.42886990

N -12.48347110 2.26723271 0.38760133

C -14.93714543 -0.55015346 0.47905692

C -16.26515286 0.23029092 0.53494472

C -16.26496473 1.58837296 0.53505000

C -14.93673371 2.36845319 0.47937882

O -14.94627323 3.62668968 0.47572492

O -14.94703802 -1.80838969 0.47516063

N -9.99020147 3.71686757 0.29359713

C -8.83172986 4.38438346 0.25270309

C -7.61850741 3.68312684 0.25350241

N -7.61891046 2.34613936 0.27837490

C -8.83124533 5.92347955 0.20317104

C -7.49726738 6.69127554 0.15650071

C -6.28399705 5.99028437 0.17660186

C -6.28459712 4.45141557 0.22461067

O -9.92054348 6.55356956 0.19977926

O -5.19521567 3.82150825 0.23887331

N -7.49702492 8.02710846 0.09536453

C -6.33845112 8.69345850 0.04744882

C -5.12512461 7.99372527 0.08511497

N -5.12539793 6.65793435 0.15233184

C -6.33590087 10.22993297 -0.05141530

C -5.12547918 10.92711124 -0.09934193

C -3.79080627 10.16120972 -0.04110612

C -3.79124196 8.76260781 0.04756649

N -7.48801877 10.90501103 -0.09330307

C -7.47696779 12.19642023 -0.19238385

C -6.26294082 12.89526637 -0.25071122

N -5.13540575 12.25977755 -0.19696397

C -8.80348893 12.97442135 -0.24768335

C -8.79294219 14.51049985 -0.37387934

C -7.61711515 15.18674582 -0.44008418

C -6.27644791 14.42852028 -0.38044733

O -9.89789714 12.35587186 -0.19007654

O -5.19191954 15.06383773 -0.43812729

N -2.63220085 10.82880799 -0.07570854

C -1.47433871 10.16097375 -0.02574228

C -1.47492950 8.76245816 0.06419395

N -2.63337290 8.09516071 0.09891611

C -0.14059236 10.92937165 -0.06747090

C 1.19232337 10.16044870 -0.01086861

C 1.19152964 8.76187436 0.07838769

C -0.14216853 7.99389938 0.12277726

N 2.35079437 10.82785832 -0.04751095

C 3.50867560 10.15968566 -0.00360465

C 3.50807037 8.76096288 0.08303833

N 2.34941137 8.09398973 0.12471690

C 4.84378765 10.92528020 -0.04948729

C 6.05359905 10.22767337 0.00412452

C 6.05533851 8.69128649 0.09886596

C 4.84171620 7.99171939 0.12876016

O -0.14295692 6.73831377 0.20666806

O -0.13991968 12.18530938 -0.14836870

N 7.21367558 8.02494599 0.15033566

C 7.21370787 6.68905385 0.20699314

C 6.00023257 5.98821106 0.22228971

N 4.84168675 6.65592633 0.19515402

N 4.85473141 12.25815600 -0.14169404

C 5.98279103 12.89318032 -0.18593010

C 7.19613710 12.19346016 -0.12523409

N 7.20609020 10.90207058 -0.02950860

C 5.99753451 14.42695853 -0.30706887

C 7.33888233 15.18446259 -0.35923923

C 8.51410895 14.50688105 -0.29530079

C 8.52324096 12.97050897 -0.17381893

C 8.54756914 5.92107073 0.25334344

C 8.54775009 4.38200866 0.29752495

C 7.33430739 3.68100411 0.29734425

C 6.00061223 4.44946766 0.26836374

N 9.70607898 3.71428363 0.33462015

C 9.70581572 2.37685024 0.35035945

C 8.49265498 1.67616820 0.33646785

N 7.33442125 2.34414832 0.32107630

O 4.91350124 15.06323435 -0.36398933

O 9.61691230 12.35085375 -0.11494079

O 4.91108035 3.81978524 0.28100927

O 9.63706928 6.55095988 0.25400554

C 11.03750752 1.60488008 0.38219822

C 11.03734525 0.20709482 0.38211366

C 9.70546654 -0.56455897 0.35027574

C 8.49247012 0.13641261 0.33640791

N 9.70541038 -1.90199439 0.33442884

C 8.54692250 -2.56944081 0.29728100

C 7.33364301 -1.86815155 0.29730705

N 7.33407783 -0.53129256 0.32100678

N 12.19898554 2.26428436 0.40964641

C 13.31423745 1.60672995 0.44342075

C 13.31407688 0.20471317 0.44335421

N 12.19866915 -0.45258090 0.40952713

C 14.65245227 2.36490227 0.48509929

C 15.98080120 1.58443775 0.52944140

C 15.98064358 0.22638526 0.52937839

C 14.65211725 -0.55377035 0.48494005

C 8.54636987 -4.10850289 0.25315034

C 7.21232348 -4.87616375 0.20676411

C 5.99901427 -4.17503859 0.22228216

C 5.99976349 -2.63629435 0.26826320

O 14.66214489 3.62313445 0.48349481

O 14.66152004 -1.81200417 0.48320990

N 7.21197024 -6.21205419 0.15012511

C 6.05346789 -6.87812665 0.09883234

C 4.84001319 -6.17826988 0.12874336

N 4.84030599 -4.84247911 0.19518308

C 6.05136286 -8.41450119 0.00394379

C 4.84138468 -9.11183903 -0.04937639

C 3.50645518 -8.34593075 -0.00344501

C 3.50618119 -6.94721235 0.08328796

N 7.20369604 -9.08916345 -0.02976787

C 7.19343749 -10.38054315 -0.12554555

C 5.97992481 -11.07998708 -0.18608546

N 4.85201376 -10.44470892 -0.14167473

C 8.52035787 -11.15788693 -0.17436514

C 8.51086234 -12.69425463 -0.29586833

C 7.33547389 -13.37156579 -0.35969032

C 5.99430350 -12.61376567 -0.30725968

O 4.91038010 -2.00635609 0.28103930

O 9.63572105 -4.73864696 0.25363115

O 4.91011811 -13.24979576 -0.36404510

O 9.61417578 -10.53847953 -0.11561861

N -2.63511148 -6.27999106 0.09939664

C -1.47682447 -6.94755618 0.06463167

C -1.47656099 -8.34608416 -0.02509810

N -2.63458031 -9.01364688 -0.07506949

C -0.14388400 -6.17931326 0.12327227

C 1.18963874 -6.94758745 0.07872671

C 1.19010372 -8.34617417 -0.01035415

C -0.14299131 -9.11478782 -0.06693897

N 2.34767675 -6.27997128 0.12502122

N 2.34841966 -9.01384478 -0.04712636

O -0.14261056 -10.37073856 -0.14765727

O -0.14438181 -4.92372122 0.20706138

N -5.13810524 -10.44402813 -0.19634493

C -6.26578229 -11.07925689 -0.25011275

C -7.47964992 -10.38011883 -0.19197773

N -7.49040689 -9.08870225 -0.09298703

C -6.27963443 -12.61251897 -0.37972389

C -7.62047459 -13.37043501 -0.43942712

C -8.79614836 -12.69390569 -0.37338637

C -8.80634693 -11.15781408 -0.24732449

O -9.90061359 -10.53900160 -0.18987921

O -5.19524992 -13.24809945 -0.43723278

H 9.43997795 15.04282753 -0.32953322

H 7.34375293 16.25116870 -0.44667495

H 9.43660551 -13.23040541 -0.33028106

H 7.34009107 -14.43827278 -0.44714289

H -7.62467400 -14.43663565 -0.53366150

H -9.72163905 -13.23047055 -0.40920626

H -17.19309382 -0.30164796 0.57395042

H -17.19275230 2.12056535 0.57422087

H -9.71831006 15.04727933 -0.40965292

H -7.62107304 16.25293839 -0.53441142

H 16.90895359 2.11648194 0.56077452

H 16.90867574 -0.30587549 0.56063628

C -0.13087455 -0.88479658 0.11631236

C 1.22555326 -1.07307652 -0.17596512

C 2.04911482 0.03323819 -0.41961458

C 1.51798418 1.32840459 -0.36328278

C 0.16110048 1.51770797 -0.06886512

C -0.66583980 0.40885031 0.16324002

H -0.75999355 -1.72958903 0.30324979

H 1.63213160 -2.06201389 -0.21405888

H 3.08372073 -0.11030268 -0.64794654

H 2.14799651 2.17364350 -0.54550239

C -0.41580104 2.94391250 0.00058899

O -1.63210432 3.12028582 0.26973011

O 0.43246150 4.06983765 -0.24072002

H 1.33026328 3.85909108 0.02853438

O -2.05510284 0.59121731 0.44604479

C -2.77696311 -0.57057095 0.02279118

O -2.22233949 -1.70050882 0.05793005

C -4.22858281 -0.44055744 -0.47377418

H -4.36425639 0.51705874 -0.92983577

H -4.43118887 -1.20918309 -1.19075051

H -4.89851498 -0.54020811 0.35366406

**PAR@TQBQ-COF**

C -8.05737467 -2.23417481 -0.35387550

C -6.84369687 -1.53349136 -0.36784321

C -5.51020220 -2.30318485 -0.39355123

C -5.51010832 -3.84295712 -0.41109600

C -6.72376385 -4.54379857 -0.40893183

C -8.05738986 -3.77410098 -0.37668665

O -9.14711185 -4.40346319 -0.36912136

O -4.42048172 -1.67384498 -0.40039330

N -4.35147513 -4.51129457 -0.42843585

C -4.35123500 -5.84871459 -0.44969830

C -5.56500172 -6.54931475 -0.45818552

N -6.72368532 -5.88126412 -0.43491123

C -3.01722634 -6.61908808 -0.46504398

C -3.01764759 -8.02041477 -0.49344092

C -4.35277860 -8.78773361 -0.51150016

C -5.56305150 -8.08850393 -0.49439529

N -9.21557670 -1.56534347 -0.32101803

C -9.21478725 -0.22763488 -0.30931730

C -8.00125409 0.47289847 -0.33463679

N -6.84329378 -0.19597886 -0.35916154

C -10.54638429 0.54444077 -0.26778260

C -10.54619317 1.94228431 -0.26765619

C -9.21440355 2.71401530 -0.30912160

C -8.00105322 2.01317755 -0.33457253

N -11.70780489 -0.11508044 -0.23115350

C -12.82282956 0.54259268 -0.18837294

C -12.82262824 1.94476829 -0.18823340

N -11.70741728 2.60212716 -0.23081962

C -14.16105109 -0.21540457 -0.13670728

C -15.48887492 0.56527985 -0.08409107

C -15.48867199 1.92285569 -0.08389294

C -14.16062100 2.70315249 -0.13637283

O -14.17039934 3.96149216 -0.13655347

O -14.17121222 -1.47373900 -0.13731848

N -9.21485474 4.05171871 -0.32052689

C -8.05649887 4.72026196 -0.35347542

C -6.84299571 4.01928090 -0.36761663

N -6.84291799 2.68176726 -0.35894637

C -8.05614668 6.26018509 -0.37619181

C -6.72234082 7.02956298 -0.40856705

C -5.50885202 6.32843136 -0.41083645

C -5.50931331 4.78865848 -0.39333295

O -9.14571523 6.88980948 -0.36841245

O -4.41974383 4.15905503 -0.40026076

N -6.72195634 8.36702256 -0.43459147

C -5.56311715 9.03480123 -0.45808158

C -4.34951984 8.33391931 -0.44974529

N -4.35006965 6.99649825 -0.42835763

C -5.56081895 10.57398031 -0.49450569

C -4.35039438 11.27292841 -0.51187966

C -3.01543759 10.50530715 -0.49389568

C -3.01533790 9.10398372 -0.46531982

N -6.71327329 11.24967698 -0.51015494

C -6.70247781 12.54424204 -0.54536976

C -5.48847136 13.24567414 -0.56285548

N -4.36065836 12.60871174 -0.54441330

C -8.02910819 13.32368221 -0.56876277

C -8.01864036 14.86422250 -0.61110240

C -6.84329597 15.54348292 -0.62756500

C -5.50240755 14.78392704 -0.60444187

O -9.12347868 12.70262657 -0.55341780

O -4.41803083 15.42222063 -0.61975826

N -1.85695469 11.17445189 -0.50578614

C -0.69820178 10.50565014 -0.48946509

C -0.69830051 9.10442418 -0.46182069

N -1.85675154 8.43550608 -0.45143770

C 0.63594258 11.27531145 -0.50086763

C 1.96976275 10.50524613 -0.48230405

C 1.96922218 9.10401472 -0.45461718

C 0.63529129 8.33444768 -0.44320748

N 3.12883736 11.17369301 -0.49246448

C 4.28702065 10.50415981 -0.47468181

C 4.28620200 9.10278757 -0.44602852

N 3.12734457 8.43472371 -0.43794372

C 5.62248080 11.27121898 -0.48606193

C 6.83239193 10.57162413 -0.46291577

C 6.83378240 9.03258717 -0.42656557

C 5.61994245 8.33218959 -0.42369236

O 0.63503198 7.07623805 -0.41847126

O 0.63620018 12.53347076 -0.52535280

N 7.99223451 8.36456088 -0.39794728

C 7.99212408 7.02723230 -0.37216377

C 6.77849055 6.32640870 -0.37925197

N 5.61993760 6.99478128 -0.40178120

N 5.63375885 12.60704013 -0.51818759

C 6.76216681 13.24326403 -0.53101750

C 7.97558950 12.54098247 -0.50810242

N 7.98531414 11.24650242 -0.47313041

C 6.77749564 14.78151413 -0.57195089

C 8.11910028 15.54005469 -0.58845421

C 9.29384363 14.85989832 -0.56681585

C 9.30292623 13.31936828 -0.52506058

C 9.32562281 6.25770887 -0.33490683

C 9.32560500 4.71789765 -0.31268972

C 8.11203478 4.01714874 -0.33083599

C 6.77855935 4.78669009 -0.36134748

N 10.48376075 4.04923640 -0.27639687

C 10.48305278 2.71160733 -0.26564452

C 9.26964523 2.01105030 -0.29434830

N 8.11170359 2.67972730 -0.32189409

O 5.69370953 15.42065937 -0.59207931

O 10.39673821 12.69743241 -0.50495107

O 5.68892100 4.15725271 -0.37192886

O 10.41524062 6.88715432 -0.32279791

C 11.81458362 1.93959791 -0.22154891

C 11.81442610 0.54178173 -0.22160960

C 10.48271462 -0.22991467 -0.26577387

C 9.26946680 0.47092545 -0.29437831

N 10.48311323 -1.56754388 -0.27677511

C 9.32479625 -2.23593148 -0.31297288

C 8.11138603 -1.53489750 -0.33097866

N 8.11137012 -0.19747831 -0.32202908

N 12.97587953 2.59916260 -0.18248311

C 14.09086288 1.94152604 -0.13829036

C 14.09071029 0.53934209 -0.13840988

N 12.97558365 -0.11804532 -0.18273018

C 15.42896415 2.69956254 -0.08464695

C 16.75673902 1.91892990 -0.03025192

C 16.75659494 0.56135032 -0.03037596

C 15.42865151 -0.21899050 -0.08488461

C 9.32444541 -3.77574518 -0.33527640

C 7.99075913 -4.54494960 -0.37245698

C 6.77729088 -3.84383729 -0.37939591

C 6.77772673 -2.30411796 -0.36145151

O 15.43906641 3.95790425 -0.08489100

O 15.43848931 -1.47733218 -0.08543171

N 7.99054583 -5.88227616 -0.39822564

C 6.83193067 -6.55002584 -0.42667739

C 5.61825631 -5.84933812 -0.42366767

N 5.61857518 -4.51192591 -0.40175496

C 6.83017232 -8.08906210 -0.46298472

C 5.62008888 -8.78837417 -0.48584717

C 4.28481400 -8.02099133 -0.47432662

C 4.28433151 -6.61962096 -0.44575632

N 7.98293488 -8.76420921 -0.47338732

C 7.97289759 -10.05868764 -0.50830664

C 6.75930564 -10.76068660 -0.53095228

N 5.63104742 -10.12420147 -0.51789062

C 9.30005208 -10.83738646 -0.52556876

C 9.29060109 -12.37791520 -0.56722221

C 8.11569294 -13.05779942 -0.58856195

C 6.77426839 -12.29894635 -0.57174517

O 5.68824019 -1.67442370 -0.37192430

O 10.41391397 -4.40544729 -0.32336986

O 5.69032801 -12.93783976 -0.59159111

O 10.39401257 -10.21570200 -0.50575120

N -1.85848750 -5.95088205 -0.45111921

C -0.70018066 -6.62006978 -0.46136528

C -0.70041171 -8.02129487 -0.48885086

N -1.85932436 -8.68982691 -0.50511759

C 0.63359454 -5.85040400 -0.44280551

C 1.96734118 -6.62029131 -0.45417686

C 1.96754763 -8.02152356 -0.48172283

C 0.63354898 -8.79127509 -0.50015752

N 3.12563334 -5.95127915 -0.43760698

N 3.12646604 -8.69024625 -0.49188653

O 0.63350777 -10.04944027 -0.52442482

O 0.63363057 -4.59219226 -0.41816741

N -4.36334404 -10.12351586 -0.54378290

C -5.49129845 -10.76023057 -0.56229278

C -6.70514615 -10.05851752 -0.54521461

N -6.71565367 -8.76394081 -0.51016123

C -5.50556884 -12.29848730 -0.60358819

C -6.84662019 -13.05774556 -0.62680308

C -8.02181703 -12.37821532 -0.61073372

C -8.03194917 -10.83766751 -0.56869504

O -9.12618359 -10.21636061 -0.55371437

O -4.42132998 -12.93702574 -0.61854205

H 10.21933419 15.39682769 -0.57879791

H 8.12468076 16.60966413 -0.61719148

H 10.21596231 -12.91505900 -0.57942981

H 8.12101741 -14.12741098 -0.61721559

H -6.85147289 -14.12735470 -0.65586020

H -8.94693676 -12.91566370 -0.62731069

H -16.41685752 0.03383823 -0.04735615

H -16.41649544 2.45456720 -0.04700698

H -8.94364142 15.40187277 -0.62756869

H -6.84791323 16.61308669 -0.65681694

H 17.68464594 2.45040543 0.00788649

H 17.68438820 0.02967482 0.00763646

C -0.92156151 -1.11421825 0.78511761

C 0.16148235 -1.73180459 0.14470115

C 1.16833393 -0.95118151 -0.43918126

C 1.09072913 0.44714189 -0.38428919

C 0.00751006 1.06429822 0.25526900

C -0.99799395 0.28367586 0.84047700

H 0.21984676 -2.79937743 0.10169584

H 1.99559582 -1.42292728 -0.92707157

O -1.94848380 -1.91033108 1.38200712

N 2.14460902 1.26811652 -0.99698958

C 3.19066132 1.54415141 -0.00186654

O 4.16919325 0.76188987 0.11868355

C 3.08722512 2.79270239 0.89344769

H 4.06937166 3.13195873 1.14765477

H 2.56754601 3.56608262 0.36728139

H 2.55188554 2.54771420 1.78713336

H -1.82415622 0.75533693 1.32958220

H -0.05175545 2.13197862 0.29698131

H -1.57431898 -2.74212019 1.68114514

H 1.75181012 2.13120028 -1.31497496

**IBU@TQBQ-COF**

C -8.51976265 -0.32063983 0.01776266

C -7.30585658 0.37981359 0.02102724

C -5.97243299 -0.39044607 0.02138008

C -5.97268854 -1.93035938 0.01677056

C -7.18648024 -2.63100945 0.01318434

C -8.52005640 -1.86070621 0.01416757

O -9.60999299 -2.48969153 0.01182836

O -4.88250339 0.23853969 0.02525753

N -4.81419537 -2.59907997 0.01604343

C -4.81414895 -3.93663840 0.01075484

C -6.02789630 -4.63737337 0.00682777

N -7.18657472 -3.96883252 0.00876818

C -3.48021985 -4.70691156 0.00879762

C -3.48046892 -6.10838341 0.00350224

C -4.81516954 -6.87612826 -0.00151149

C -6.02565350 -6.17719998 0.00007732

N -9.67812553 0.34856828 0.01750900

C -9.67733785 1.68625615 0.01820788

C -8.46343215 2.38671535 0.02112062

N -7.30527721 1.71750910 0.02361212

C -11.00953949 2.45834135 0.01551490

C -11.00938103 3.85610092 0.01549619

C -9.67700063 4.62788046 0.01821841

C -8.46325845 3.92713977 0.02112219

N -12.17147701 1.79876244 0.01294231

C -13.28734114 2.45636296 0.00960580

C -13.28718070 3.85859430 0.00961061

N -12.17116509 4.51594200 0.01294259

C -14.62650449 1.69837451 0.00556015

C -15.95540895 2.47897765 0.00136924

C -15.95525401 3.83658445 0.00134831

C -14.62617288 4.61688623 0.00552728

O -14.63596078 5.87524969 0.00562110

O -14.63657675 0.44001487 0.00563932

N -9.67747782 5.96557304 0.01751301

C -8.51896138 6.63451193 0.01773468

C -7.30521857 5.93377637 0.02104500

N -7.30495311 4.59608001 0.02364254

C -8.51889323 8.17458086 0.01410818

C -7.18513702 8.94456946 0.01316906

C -5.97151087 8.24363185 0.01680897

C -5.97161858 6.70371970 0.02143721

O -9.60868609 8.80382026 0.01179797

O -4.88183499 6.07448149 0.02535950

N -7.18491017 10.28239235 0.00874699

C -6.02607059 10.95066117 0.00680999

C -4.81249465 10.24963274 0.01075245

N -4.81286070 8.91207825 0.01614655

C -6.02345660 12.49048422 0.00000057

C -4.81279802 13.18912363 -0.00160168

C -3.47828637 12.42105092 0.00348350

C -3.47837441 11.01958407 0.00880959

N -7.17556680 13.16712851 -0.00456215

C -7.16382802 14.46232729 -0.01121254

C -5.94928226 15.16314118 -0.01288104

N -4.82209878 14.52517295 -0.00769548

C -8.48972889 15.24331019 -0.01743573

C -8.47789612 16.78446220 -0.02562047

C -7.30200508 17.46294190 -0.02729061

C -5.96183559 16.70188533 -0.02099273

O -9.58465913 14.62307152 -0.01589208

O -4.87686784 17.33939559 -0.02248071

N -2.31999314 13.09018527 0.00276626

C -1.16149825 12.42121082 0.00657430

C -1.16160120 11.01972366 0.01082222

N -2.31999955 10.35079385 0.01213846

C 0.17232304 13.19103566 0.00635332

C 1.50592223 12.42082853 0.01035418

C 1.50564577 11.01933799 0.01332421

C 0.17191239 10.24959291 0.01390774

N 2.66458792 13.08948250 0.01108647

C 3.82269052 12.42004255 0.01498748

C 3.82243199 11.01856732 0.01690519

N 2.66387271 10.35007823 0.01578275

C 5.15736022 13.18781147 0.01745710

C 6.36785662 12.48892003 0.02163747

C 6.37012403 10.94908172 0.02343209

C 5.15638774 10.24829737 0.02051578

O 0.17172706 8.99119332 0.01695852

O 0.17251080 14.44943494 0.00295413

N 7.52881568 10.28057007 0.02788286

C 7.52876556 8.94274272 0.02945626

C 6.31499990 8.24204150 0.02520279

N 5.15647834 8.91072256 0.02096918

N 5.16692722 14.52385913 0.01576319

C 6.29422432 15.16161248 0.01844968

C 7.50862634 14.46056401 0.02276114

N 7.52011082 13.16534088 0.02413106

C 6.30706015 16.70036894 0.01692801

C 7.64736284 17.46120434 0.02021073

C 8.82312591 16.78250448 0.02437030

C 8.83466873 15.24132224 0.02590435

C 8.86236134 8.17248766 0.03598692

C 8.86213140 6.63241399 0.03750256

C 7.64825421 5.93192082 0.03173669

C 6.31481067 6.70212456 0.02547371

N 10.02050752 5.96323315 0.04438356

C 10.01974696 4.62553129 0.04565815

C 8.80586990 3.92505789 0.03849470

N 7.64771797 4.59423048 0.03174108

O 5.22220794 17.33806625 0.01304248

O 9.92947312 14.62087652 0.02974073

O 5.22491106 6.07308994 0.02064274

O 9.95226586 8.80151920 0.04014366

C 11.35193289 3.85344211 0.05518471

C 11.35176684 2.45567002 0.05519270

C 10.01940609 1.68388841 0.04569616

C 8.80568847 2.38464544 0.03852436

N 10.01985449 0.34618272 0.04436279

C 8.86131994 -0.32272286 0.03745794

C 7.64761098 0.37805644 0.03167408

N 7.64738393 1.71574840 0.03173159

N 12.51384935 4.51301442 0.06362597

C 13.62968255 3.85540803 0.07313818

C 13.62951919 2.45317505 0.07312567

N 12.51353196 1.79582933 0.06363833

C 14.96881411 4.61339537 0.08459825

C 16.29766083 3.83277960 0.09621295

C 16.29750529 2.47518651 0.09621793

C 14.96847555 1.69487911 0.08461624

C 8.86118993 -1.86279892 0.03598166

C 7.52740855 -2.63274029 0.02945574

C 6.31381174 -1.93175320 0.02516215

C 6.31398281 -0.39183393 0.02539034

O 14.97891774 5.87175451 0.08453953

O 14.97828923 0.43651917 0.08454632

N 7.52714809 -3.97056886 0.02790138

C 6.36829429 -4.63880344 0.02341914

C 5.15472289 -3.93772834 0.02044478

N 5.15512742 -2.60015510 0.02088301

C 6.36566092 -6.17864537 0.02171757

C 5.15498880 -6.87725261 0.01751617

C 3.82050513 -6.10915646 0.01494409

C 3.82058451 -4.70767639 0.01682028

N 7.51775737 -6.85533294 0.02428977

C 7.50595019 -8.15055431 0.02295997

C 6.29137108 -8.85131568 0.01861780

N 5.16422165 -8.21330246 0.01589489

C 8.83180233 -8.93163709 0.02618854

C 8.81988895 -10.47281897 0.02477183

C 7.64396271 -11.15123540 0.02058815

C 6.30384119 -10.39007560 0.01718151

O 5.22423312 0.23745460 0.02054710

O 9.95094457 -2.49208483 0.04018146

O 5.21883819 -11.02751319 0.01332063

O 9.92675475 -8.31145840 0.03010076

N -2.32167991 -4.03840556 0.01199216

C -1.16344568 -4.70762141 0.01065529

C -1.16368322 -6.10910837 0.00649774

N -2.32234188 -6.77780364 0.00271255

C 0.17025362 -3.93781447 0.01374325

C 1.50379965 -4.70788557 0.01323221

C 1.50373841 -6.10937561 0.01029747

C 0.16995177 -6.87926108 0.00630796

N 2.66218352 -4.03890149 0.01563722

N 2.66224475 -6.77831761 0.01102477

O 0.16982756 -8.13765621 0.00290661

O 0.17037996 -2.67941743 0.01674165

N -4.82479586 -8.21217586 -0.00759024

C -5.95213229 -8.84987366 -0.01263583

C -7.16650896 -8.14876378 -0.01092319

N -7.17792853 -6.85356302 -0.00440697

C -5.96506365 -10.38861203 -0.02064025

C -7.30541508 -11.14934575 -0.02682431

C -8.48114505 -10.47057659 -0.02513245

C -8.49259871 -8.92942459 -0.01703264

O -9.58737855 -8.30891566 -0.01552279

O -4.88024831 -11.02638850 -0.02219987

H 9.74773271 17.32102052 0.02662839

H 7.65122066 18.53119844 0.01920028

H 9.74436775 -11.01155962 0.02712594

H 7.64756216 -12.22123418 0.01966222

H -7.30934038 -12.21932111 -0.03253202

H -9.40578246 -11.00902275 -0.02951792

H -16.88408405 1.94750959 -0.00153572

H -16.88381070 4.36826428 -0.00154201

H -9.40240559 17.32313244 -0.03004420

H -7.30566726 18.53291911 -0.03301791

H 17.22630804 4.36424277 0.10429145

H 17.22602795 1.94350960 0.10429307

C -0.06879754 1.35203657 0.06871139

C 1.30380916 1.58356885 0.22954911

C 1.81334392 2.88374408 0.11390691

C 0.95021785 3.95266888 -0.16179268

C -0.42290839 3.72145423 -0.32131378

C -0.93235747 2.42093797 -0.20620882

H 1.96287200 0.76746643 0.44060072

H 2.86149747 3.06032497 0.23620560

H -1.08220157 4.53775879 -0.53114374

H -1.98047360 2.24410552 -0.32832843

C 1.51134438 5.38103522 -0.28950390

C 1.50165329 6.06220827 1.09181030

C 2.95523505 5.31889258 -0.82174512

H 1.89153181 7.05468814 1.00266074

H 0.49843831 6.10491861 1.46130399

H 2.10831639 5.50047723 1.77110595

O 3.41061653 6.26364580 -1.51717642

O 3.77834392 4.18749622 -0.52584909

H 4.69791141 4.46087258 -0.49143633

C -0.62711213 -0.07759464 0.19423956

C -2.07273371 -0.02105502 0.72149558

C -2.94567237 0.78531472 -0.25804280

C -2.62870350 -1.45181359 0.84690055

H -2.55899122 1.77920638 -0.34529833

H -2.93444127 0.31265857 -1.21792392

H -3.95015022 0.82438070 0.10855215

H -2.02209755 -2.01162224 1.52775920

H -3.63334847 -1.41343024 1.21310687

H -2.61681017 -1.92453544 -0.11295504

H -0.61614462 -0.55010973 -0.76566880

H -0.02118735 -0.63825644 0.87486746

H -2.08425645 0.45163755 1.68137256

H 0.90490876 5.94331341 -0.96848383
